# METTL3 facilitates tumor progression via an m^6^A-IGF2BP2-dependent mechanism in colorectal carcinoma

**DOI:** 10.1186/s12943-019-1038-7

**Published:** 2019-06-24

**Authors:** Ting Li, Pei-Shan Hu, Zhixiang Zuo, Jin-Fei Lin, Xingyang Li, Qi-Nian Wu, Zhan-Hong Chen, Zhao-Lei Zeng, Feng Wang, Jian Zheng, Demeng Chen, Bo Li, Tie-Bang Kang, Dan Xie, Dongxin Lin, Huai-Qiang Ju, Rui-Hua Xu

**Affiliations:** 1 State Key Laboratory of Oncology in South China, Collaborative Innovation Center for Cancer Medicine, Sun Yat-sen University Cancer Center, 651 Dongfeng East Road, Guangzhou, 510060 People’s Republic of China; 2 Department of Medical Oncology, Sun Yat-sen University Cancer Center, Guangzhou, 510060 China; 3 Department of Pathology, Sun Yat-sen University Cancer Center, Guangzhou, 510060 China; 4 Department of Medical Oncology and Guangdong Key Laboratory of Liver Disease, the Third Affiliated Hospital of Sun Yat-sen University, Guangzhou, 510060 China; 5 Center for Translational Medicine, The First Affiliated Hospital, Sun Yat-sen University, Guangzhou, 510080 China; 6 Department of Biochemistry and Molecular Biology, Zhongshan School of Medicine, Sun Yat-Sen University, Guangzhou, 510080 China; 7 State Key Laboratory of Molecular Oncology, Chinese Academy of Medical Science and Peking Union Medical College, Beijing, 100021 China

**Keywords:** Colorectal cancer, N^6^-methyladenosine (m^6^A), METTL3, SOX2, IGF2BP2

## Abstract

**Background:**

Colorectal carcinoma (CRC) is one of the most common malignant tumors, and its main cause of death is tumor metastasis. RNA N^6^-methyladenosine (m^6^A) is an emerging regulatory mechanism for gene expression and methyltransferase-like 3 (METTL3) participates in tumor progression in several cancer types. However, its role in CRC remains unexplored.

**Methods:**

Western blot, quantitative real-time PCR (RT-qPCR) and immunohistochemical (IHC) were used to detect METTL3 expression in cell lines and patient tissues. Methylated RNA immunoprecipitation sequencing (MeRIP-seq) and transcriptomic RNA sequencing (RNA-seq) were used to screen the target genes of METTL3. The biological functions of METTL3 were investigated in vitro and in vivo. RNA pull-down and RNA immunoprecipitation assays were conducted to explore the specific binding of target genes. RNA stability assay was used to detect the half-lives of the downstream genes of METTL3.

**Results:**

Using TCGA database, higher METTL3 expression was found in CRC metastatic tissues and was associated with a poor prognosis. MeRIP-seq revealed that SRY (sex determining region Y)-box 2 (SOX2) was the downstream gene of METTL3. METTL3 knockdown in CRC cells drastically inhibited cell self-renewal, stem cell frequency and migration in vitro and suppressed CRC tumorigenesis and metastasis in both cell-based models and PDX models. Mechanistically, methylated *SOX2* transcripts, specifically the coding sequence (CDS) regions, were subsequently recognized by the specific m^6^A “reader”, insulin-like growth factor 2 mRNA binding protein 2 (IGF2BP2), to prevent *SOX2* mRNA degradation. Further, SOX2 expression positively correlated with METTL3 and IGF2BP2 in CRC tissues. The combined IHC panel, including “writer”, “reader”, and “target”, exhibited a better prognostic value for CRC patients than any of these components individually.

**Conclusions:**

Overall, our study revealed that METTL3, acting as an oncogene, maintained SOX2 expression through an m^6^A-IGF2BP2-dependent mechanism in CRC cells, and indicated a potential biomarker panel for prognostic prediction in CRC.

**Electronic supplementary material:**

The online version of this article (10.1186/s12943-019-1038-7) contains supplementary material, which is available to authorized users.

## Background

Colorectal carcinoma (CRC) is a highly lethal cancer with an increasing incidence worldwide [1]. Despite therapeutic advances over the past few decades, the mortality rate of CRC remains high, which is mainly ascribed to recurrence and distant organ metastasis [2]. Recent studies, including ours, have indicated that a small population of cancer cells, called cancer stem-like cells (CSCs), display increased self-renewal ability, and cause chemotherapy resistance, which are possible mechanism for tumor recurrence and metastasis [3–5]. Therefore, exploring the factors that drive tumor initiation and establishing a more accurate model for prognostic prediction in CRC are urgently needed.

Epigenetic regulatory mechanisms, such as DNA methylation, or N^6^-methyladenosine (m^6^A), are emerging research frontiers in tumor biology [6–9]. As the most abundant post-transcriptional modification, m^6^A modification is mainly mediated by m^6^A WERs (“writers”, “erasers” and “readers”), and is reported to be related to RNA fate control through influencing alternative polyadenylation and pre-mRNA splicing, as well as regulating RNA stability and translation efficiency [10–13]. Our previous study also demonstrated that one of the RNA demethylases, fat-mass and obesity-associated protein (FTO), plays a critical role in cell transformation in leukemia cells [14]. These findings have indicated that m^6^A has a broad impact on embryonic development, circadian clock control, and the DNA damage response, as well as on tumor progression [10, 14–17]. Furthermore, it is worth noting that these impressive biological functions rely on the target genes of the m^6^A “writers” or “erasers”, and the fate of the target transcripts generally rely on the specific recognition of m^6^A “readers” [18]. As a reversible epi-transcriptome modulator, methyltransferase-like 3 (METTL3) is a key member of the m^6^A methyltransferase complex, and has recently been reported to be essential for tumor progression in leukemia, hepatocellular carcinoma, and malignant glioma via diverse downstream genes [16, 17, 19]. However, the functions of m^6^A modification and the underlying connection among the m^6^A “writers”, “readers”, and “targets” are still unexplored in CRC.

Here, we first demonstrated the function of METTL3 in facilitating CRC progression, and identified *SRY (sex determining region Y)-box 2 (SOX2)* as the downstream target of METTL3. Moreover, insulin-like growth factor 2 mRNA binding protein 2 (IGF2BP2) was indicated to prolong the *SOX2* life-span. Overall, our study reveals that METTL3 is a promising biomarker for prognostic prediction and a potential therapeutic target in CRC.

## Methods

### Tissue specimens and patient information

A total of 432 paraffin-embedded, archived CRC specimens and paired adjacent normal tissue samples, including 43 matched liver metastasis tissues and 52 matched lymph node metastasis tissues, were obtained at the SYSUCC (Guangzhou, China) between January 2010 and July 2013 as previously described [20]. The clinical CRC specimens were collected with permission from our Institutional Research Ethics Committee. The clinical characteristics of the samples are summarized in Additional file 1: Table S1.

### Methylated RNA immunoprecipitation sequencing (MeRIP-seq)

MeRIP-seq was conducted in accordance with a previously reported protocol with minor modifications [21]. Briefly, 50 μg of total RNA was extracted and purified using RiboMinus™ Eukaryote Kit v2 (A15020, Invitrogen) to deplete the ribosomal RNA from the total RNA. Next, RNA Fragmentation Reagents (AM8740, Invitrogen) were used to shear the RNA into approximately 100-nt fragments. Approximately 1/10 of the fragmented RNA was saved as the input control for further RNA sequencing by RiboBio (Guangzhou, China). The remaining were incubated with an anti-m^6^A antibody (202,203, Synaptic Systems) for one hour at 4 °C, and then mixed with prewashed Pierce™ Protein A/G Magnetic Beads (88,803, Thermo Scientific) in immunoprecipitation buffer at 4 °C overnight. The m^6^A antibody was digested with proteinase K digestion buffer and the methylated RNA was purified for further MeRIP sequencing by RiboBio (Guangzhou, China).

### MeRIP-qPCR

M^6^A modifications of individual genes were determined using MeRIP-qPCR assay. Briefly, poly(A) RNA was first purified from 50 μg of total RNA using the Dynabeads™ mRNA Purification Kit (61,006, Invitrogen) and one-tenth of the RNA was saved as the input control. Pierce™ Protein A/G Magnetic Beads (88,803, Thermo Scientific) were prewashed and incubated with 5 μg of anti-m^6^A antibody (202,003, Synaptic Systems) or rabbit IgG for 2 h at 4 °C with rotation. After 3 washes, the antibody-conjugated beads were mixed with purified poly(A) RNA, and 1 × immunoprecipitation buffer supplemented with RNase inhibitors. Then, the methylated mRNAs were precipitated with 5 mg of glycogen and one-tenth volumes of 3 M sodium acetate in a 2.5 volume of 100% ethanol at − 80 °C overnight after proteinase K digestion. Further enrichment was calculated by qPCR and the corresponding m^6^A enrichment in each sample was calculated by normalizing to the input.

### RNA pull-down assays

RNA was first transcribed by the MEGAscript T7 Transcription Kit (AM1334, Thermo Scientific). Then, the amplified RNA was end-labeled with desthiobiotin by using Pierce RNA 3′ End Desthiobiotinylation Kit (20,163, Thermo Scientific). Finally, RNA pull-down assays were performed using the Pierce Magnetic RNA-Protein Pull-Down Kit (20,164, Thermo Scientific). Up to 50 pmol of biotinylated RNAs was mixed with 2 mg of protein lysates and 50 μl of streptavidin beads. After incubation and three washes, the streptavidin beads were boiled and used for the immunoblotting assay.

### RNA immunoprecipitation (RIP) assays

RIP was conducted with the Magna RIP RNA-Binding Protein Immunoprecipitation Kit (17–700, Millipore) according to the manufacturer’s instructions. Briefly, magnetic beads coated with 5 μg of specific antibodies against mouse immunoglobulin G (17–700, Millipore), or IGF2BP2 (ab#128175, Abcam) were incubated with prepared cell lysates overnight at 4 °C. Then, the RNA-protein complexes were washed 6 times and incubated with proteinase K digestion buffer. RNA was finally extracted by phenol-chloroform RNA extraction methods. The relative interaction between IGF2BP2 and *SOX2* transcripts was determined by qPCR and normalized to the input.

### Vector and m^6^A mutation assays

The potential m^6^A sites were predicted using an online tool, SRAMP (http://www.cuilab.cn/sramp/). Full-length *SOX2* transcripts, the *SOX2* CDS region, the *SOX2* three prime untranslated region (3′-UTR), and the m^6^A motif depleted CDS or 3′-UTR regions were cloned into pcDNA3.1 for the RNA pull down assay. The specific sequences are shown in Additional file 2: Table S2.

### RNA stability assays

CRC cells were seeded in 12-well plates overnight, and then treated with actinomycin D (5 μg/mL, HY-17559, MedChemExpress) at the 0, 3, 6 h. Total RNA was then isolated by TRIzol (15,596,018, Invitrogen) and analyzed by qPCR. The mRNA expression for each group at the indicated time was calculated and normalized by β-Actin. The mRNA half-lives time were estimated according to the linear regression analysis.

### Statistical analysis

All data and error bars are presented as the mean ± SDs from at least three independent experiments. All differences between two independent groups were evaluated by a two-tailed Student’s *t*-test. Survival curves were generated using the Kaplan–Meier method and compared using the log-rank test. Survival data were evaluated by univariate and multivariate Cox regression analyses. To investigate the correlation between two independent groups, the Pearson’s *Chi*-square test was used. The MedCalc software was used to generate the ROC curve, and the data were analyzed by two-tailed *t* test. The indicated *P* values (*P < 0.05 and **P < 0.01) were considered statistically significant.

Additional Materials and Methods are described in Additional file 3.

## Results

### METTL3 is highly expressed in metastatic CRC and associated with poor prognosis

**Fig. 1:**
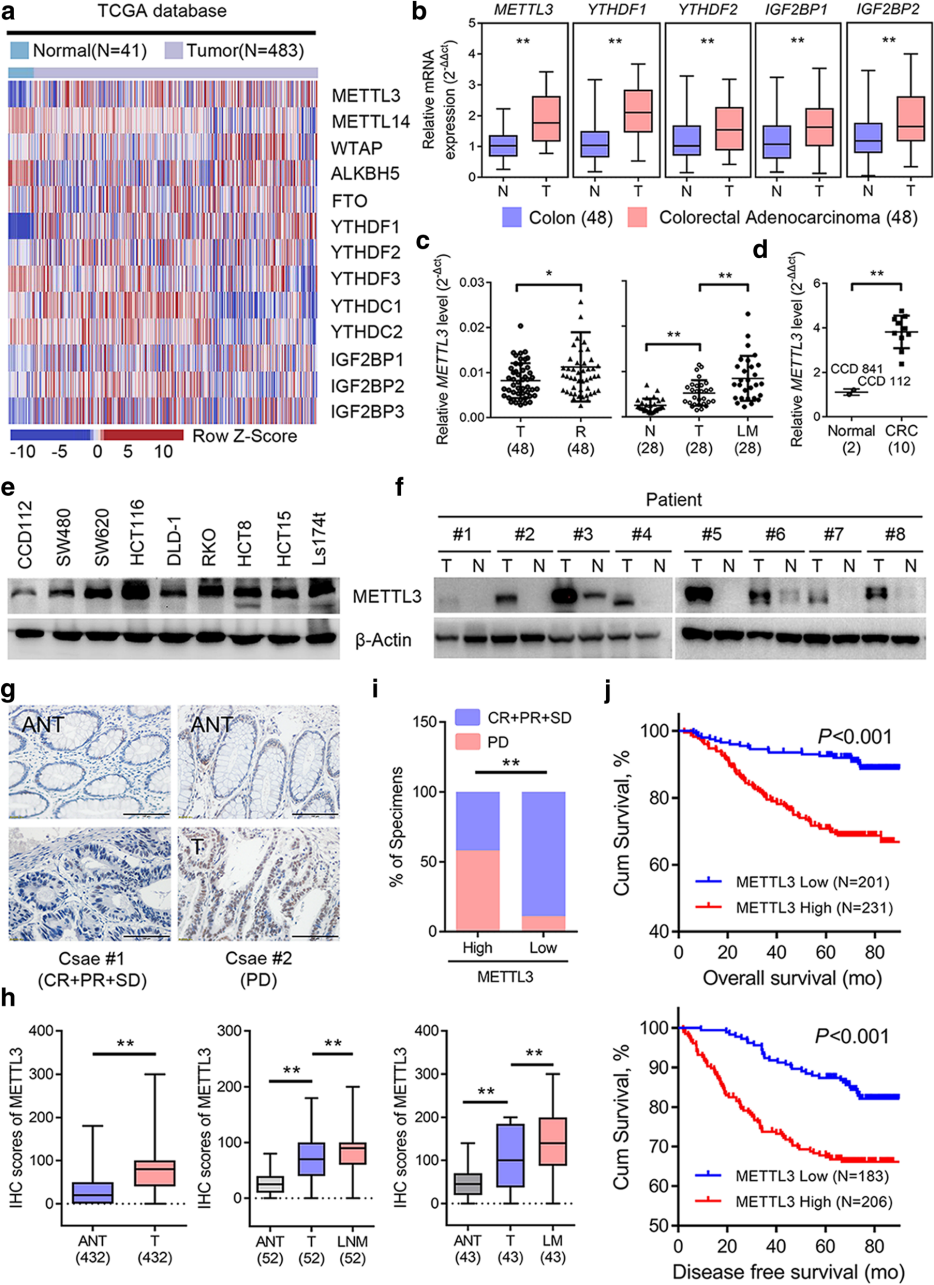
METTL3 is highly expressed in metastatic CRC and associated with poor prognosis. **a** Heat map profiling the expression of m^6^A WERs in the TCGA database of COAD. **b** Real-time PCR analysis of m^6^A WER expression in 48 paired CRC tumor tissues (T) and adjacent normal tissues (N). **c** Real-time PCR analysis of METTL3 expression in CRC tissues from patients with recurrence (R, *n* = 48) and without recurrence (T, *n* = 48), 28 paired liver metastatic tissues (LM) versus primary tumor tissues (T), and adjacent normal tissues (N). **d-e** Real-time PCR analysis and Immunoblotting assay of METTL3 expression in normal colonic epithelial cell lines and CRC cell lines. **f** Immunoblotting assay of METTL3 expression in eight paired CRC primary tumor samples (T) and adjacent normal tissues (N). **g** Representative images showing METTL3 expression in CRC adjacent normal tissues (ANT) (upper) versus high METTL3 expression in CRC tumor tissues (T) (lower) (scale bar: 100 μm). **h** METTL3 IHC staining scores in CRC tumor tissues versus ANT (*n* = 432), paired lymph node metastatic tissues (LNM, *n* = 52) or paired liver metastatic tissues (LM, *n* = 43). **i** Correlation between METTL3 expression with CRC patient response to FOLFOX or XELOX chemotherapy. The data were analyzed by Pearson’s *Chi-*square test. **j** Kaplan-Meier analysis of OS time (upper) and DFS time (lower) based on METTL3 expression. CR, complete response; PR, partial response; SD, stable disease; PD, progressive disease. The data in **b, c, d**, and **i**, are presented as the mean ± SDs (*n* = 3). *P < 0.05, **P < 0.01 (Student’s *t*-test). β-Actin was used as the loading control

To evaluate the expression profile of m^6^A WERs in CRC, we analyzed The Cancer Genome Atlas (TCGA) database, and the results showed that several m^6^A WERs were dysregulated in colon adenocarcinoma (COAD) (Fig. 1a). We next verified that METTL3, YTH N^6^-methyladenosine RNA binding protein 1 (YTHDF1), YTH N^6^-methyladenosine RNA binding protein 2 (YTHDF2), insulin like growth factor 2 mRNA binding protein 1 (IGF2BP1), and IGF2BP2 were significantly increased in CRC tumors tissues from Sun Yat-sen University Cancer Center (SYSUCC), while the other WERs showed no significant differences (Fig. 1b and Additional file 4: Figure S1a). Additionally, METTL3 was commonly highly expressed in most human cancers from TCGA database (Additional file 4: Figure S1b). These expression differences of METTL3 prompted us to investigate its functional and clinical consequences in CRC. Further validation showed that METTL3 was consistently elevated in recurrent CRC tissues and metastatic liver tissues (Fig. 1c). The METTL3 mRNA and protein level in CRC cell lines were also increased relative to the normal colonic epithelial cell lines (Figs. 1d-e). Moreover, METTL3 protein levels were notably increased in representative CRC patient tissues compared with normal tissue (Fig. 1f). To investigate the clinical implication of METTL3 with CRC, we performed IHC staining for METTL3 in our archived CRC tissue microarray, described previously [20]. Our results indicated that METTL3 staining was increased in primary CRC tissues compared with the adjacent normal tissue. Similarly, the significant elevation of METTL3 was also observed in matched lymph node and liver metastatic foci (Figs. 1g-h). We next explored the correlation between METTL3 with the disease control rate in CRC patients, and found that patients with high METTL3 expression had a poorer benefit from standard chemotherapy (Fig. 1i). Moreover, the CRC patients with high METTL3 expression had both shorter overall survival (OS) and disease-free survival (DFS) (Fig. 1j), which suggests that METTL3 expression might serve as a prognostic marker for OS and DFS in CRC patients.

### *SOX2* is regulated by METTL3-mediated m^6^A modification

**Fig. 2:**
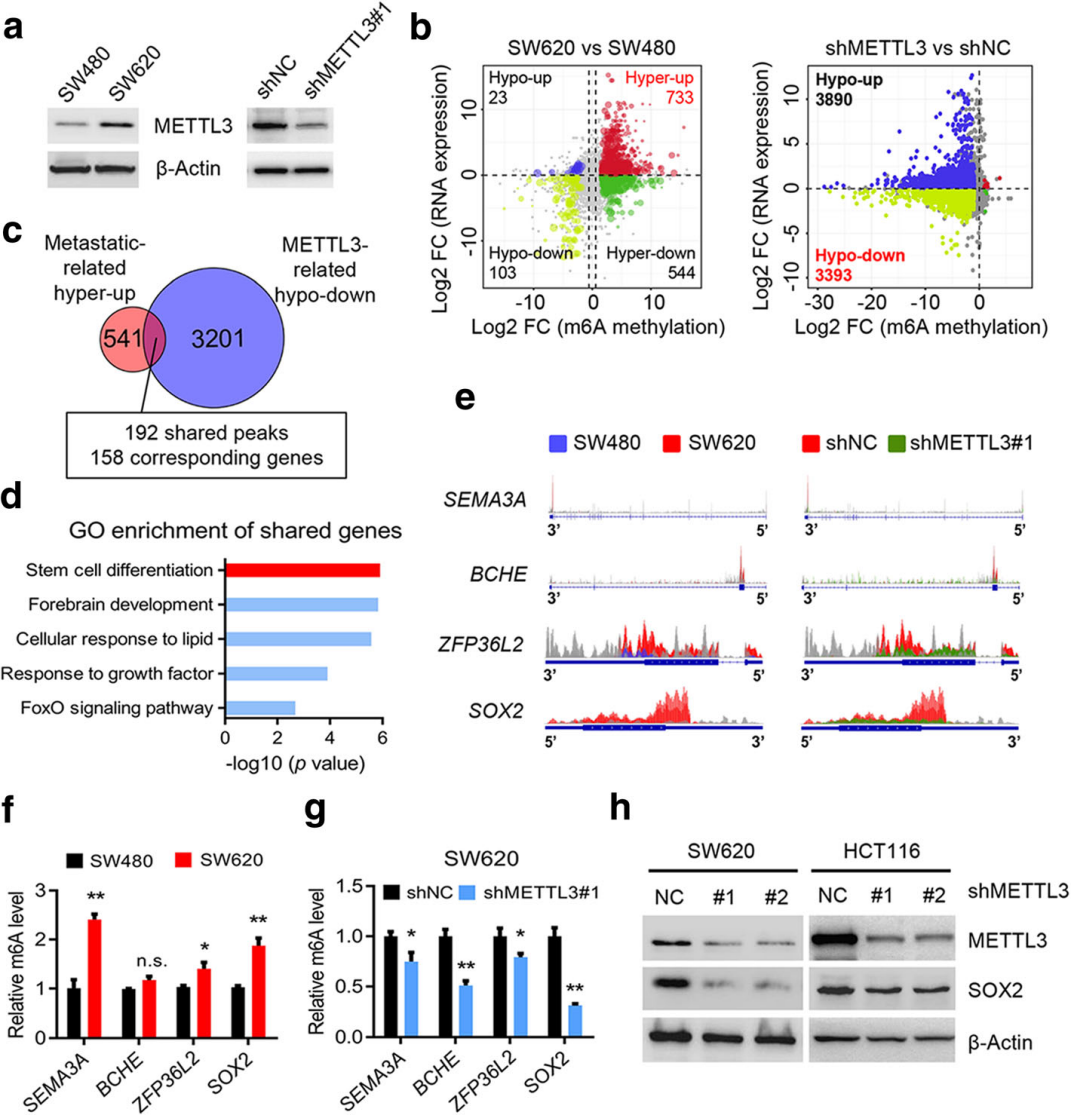
Identification of METTL3 targets via MeRIP-seq and RNA-seq. **a,** Immunoblotting of METTL3 in SW480 and SW620 cells (left), and in METTL3 knockdown SW620 and control SW620 cells (right). **b,** Distribution of peaks (fold change > 1.5 or < − 1.5, *P* < 0.05) with a significant change in both the RNA expression level and m^6^A level in SW620 cells compared with SW480 cells (left), and in METTL3 knockdown SW620 cells compared to control SW620 cells (right). **c,** Venn diagram showing the shared peaks between metastatic-related hyper-up peaks and METTL3-related hypo-down peaks. A total of 192 shared peaks corresponding to 158 specific genes were observed. **d,** GO biological process enrichment analysis of the above shared peaks. **e,** The m^6^A abundances in *SEMA3A, BCHE, ZFP36L2,* and *SOX2* transcripts in SW620 cells related to the SW480 cells (left panel), and in METTL3-knockdown SW620 cells (shMETTL3#1) related to the control SW620 cells (shNC) (right panel). **f,** Gene-specific m^6^A qPCR analysis of alterations in the m^6^A level in four representative genes in SW620 and SW480 cells. **g,** Gene-specific m^6^A qPCR analysis of alterations in the m^6^A level in four representative genes in METTL3-knockdown SW620 and control SW620 cells. **h,** Immunoblotting assay of SOX2 after METTL3-knockdown in SW620 and HCT116 cells. The data in f, and g are presented as the mean ± SDs (*n* = 3). *P < 0.05, **P < 0.01 (Student’s *t*-test). β-Actin was used as the loading control. The relative m^6^A level was normalized by input. The relative expression level was normalized by the β-Actin

To investigate the potential role of METTL3 in tumor progression, we firstly noted that METTL3 was elevated in SW620 cells compared with SW480 cells (Fig. 2a), a pair of cell lines isolated from abdominal metastatic foci and the primary tumor, respectively, of a single patient. The two cell lines exhibit different metastatic abilities [22], which points to the connection between METTL3 and metastasis. Therefore, we performed MeRIP-seq and RNA-seq in SW480, SW620 and METTL3 knockdown SW620 cells. The results showed that there were generally hyper-methylated peaks in SW620 cells compared with the SW480 cells, and the methylation level of the identified peaks in SW620 cells was downregulated after METTL3 knockdown (Additional file 5: Figures S2a-b). We further investigated the mRNA expression, corresponding to each peak, in our RNA-seq data, and described the distribution of peaks with a significant change in both the RNA level and the m^6^A level. We found 733 hyper-methylated m^6^A peaks with higher mRNA expression in SW620 cells versus SW480 cells, and thereafter called these peaks metastatic-related hyper-up peaks. Similarly, we found 3393 hypo-methylated m^6^A peaks with lower mRNA expression in METTL3-knockdown SW620 cells relative to control SW620 cells, and named these peaks METTL3-related hypo-down peaks (Fig. 2b). Focusing on the peaks in these two groups, we found that 192 specific peaks, corresponding to 158 genes, were shared (Fig. 2c). Interestingly, we found that the shared genes were the most enriched in the stem cell differentiation pathway through GO enrichment analysis on metascape website, indicating that this pathway might be regulated by METTL3 and promoted tumor metastasis via an m^6^A mechanism (Fig. 2d).

We next screened the genes listed in the stem cell differentiation pathway in our m^6^A-seq data, and found four genes, *semaphorin 3A (SEMA3A)*, *butyrylcholinesterase (BCHE)*, *ZFP36 ring finger protein like 2 (ZFP36L2)*, and *SOX2*, that exhibited a substantial increase in m^6^A level in SW620 cells compared with SW480 cells and showed a consistent decreased m^6^A level in METTL3-knockdown SW620 cells compared with control cells (Fig. 2e). Gene-specific m^6^A pull down assay and qPCR analysis showed that the m^6^A levels of *SOX2, ZFP36L2,* and *SEMA3A* were increased in SW620 cells compared with SW480 cells (Fig. 2f). However, *SOX2* exhibited the most consistent decreased m^6^A level and mRNA level in METTL3 knockdown CRC cells versus the control cells (Fig. 2g, and Additional file 5: Figures S2c-d). Moreover, the significant decreased protein level of SOX2 was detected after METTL3 inhibition in SW620 and HCT116 cells (Fig. 2h). Considering that SOX2 is considered the important CSC marker to promote tumor initiation and participate in tumor metastasis [23, 24], we presumed that METTL3 promoted CRC stemness and metastasis in an m^6^A-dependent manner to maintain *SOX2* expression.

### METTL3 promotes CRC cell stemness in vitro

**Fig. 3:**
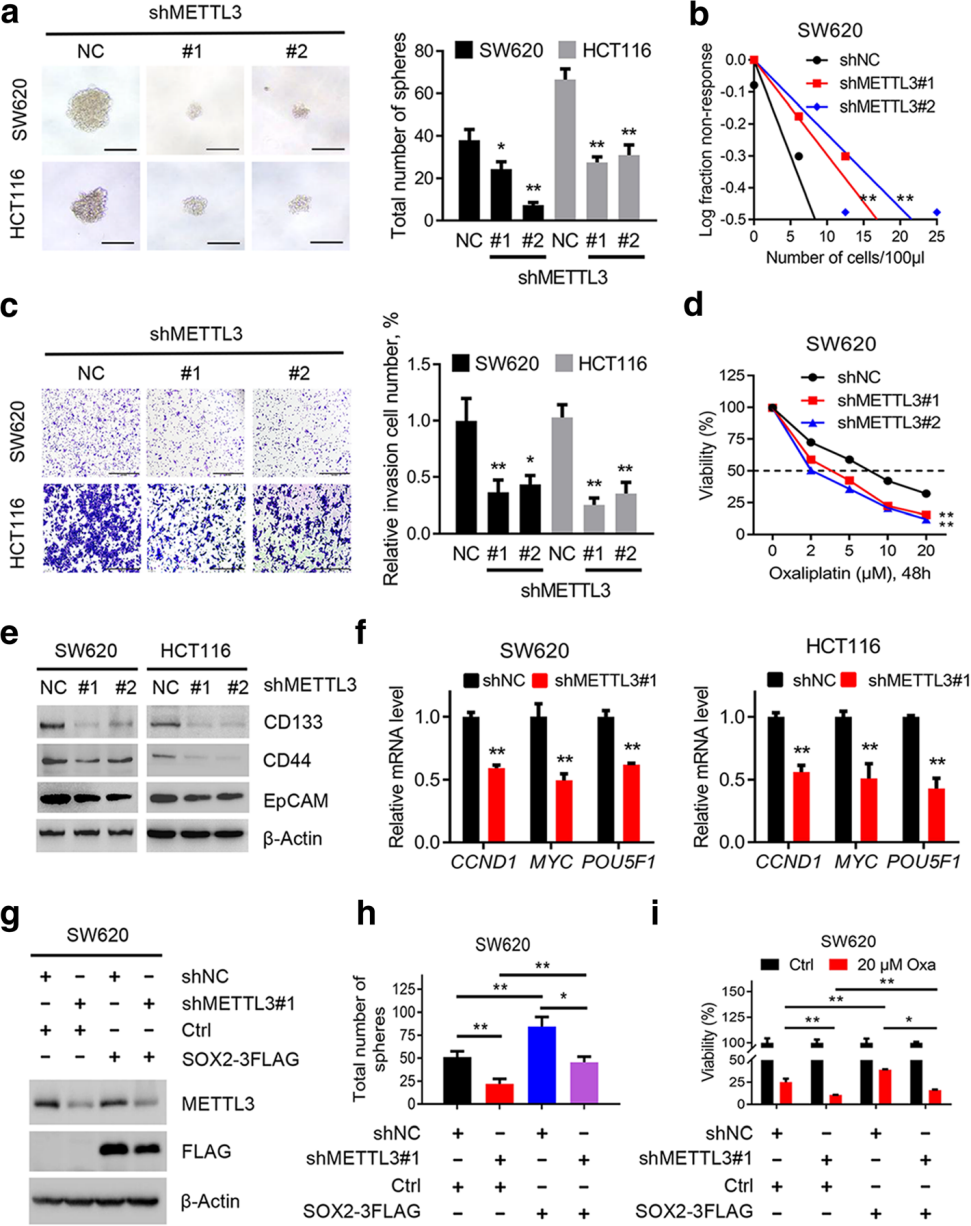
METTL3 promotes CRC cell stemness in vitro. **a,** Representative images and quantification of the in vitro sphere-formation assay of METTL3 knockdown CRC cells and control cells (*n* = 6). Scale bar: 200 μm. **b,** In vitro limiting dilution assay of METTL3 knockdown and control SW620 cells. A well not containing spheres (diameter ≥ 50 μm) was defined as a non-response (*n* = 12). **c,** Representative images and quantification of invaded METTL3-knockdown and control SW620 and HCT116 cells. Scale bar: 100 μm. **d,** Cell viability of METTL3-knockdown SW620 cells and control SW620 cells after treatment with oxaliplatin for 48 h. **e,** Immunoblotting analysis of stem-like cell surface antigen (CD133, CD44, and EpCAM) in METTL3-knockdown and control SW620 and HCT116 cells. **f,** Real-time PCR analysis of SOX2 targets genes (*CCND1, MYC,* and *POU5F1*) in METTL3-knockdown and control SW620 and HCT116 cells. **g,** Immunoblotting analysis of SOX2 and METTL3 in METTL3-knockdown and control SW620 cells with or without SOX2 overexpression. **h,** Quantification of the in vitro sphere-formation assay of METTL3 knockdown and control SW620 cells with or without SOX2 overexpression. (n = 6). **i,** Cell viability of METTL3-knockdown and control SW620 cells with or without SOX2 overexpression after oxaliplatin treatment for 48 h. All data are presented as the mean ± SDs (n = 3). *P < 0.05, **P < 0.01 (Student’s *t*-test). β-Actin was used as the loading control. The relative expression level was normalized by β-Actin

We next performed several experiments to test our hypothesis. Interestingly, a decrease in sphere numbers and sizes as well as a markedly reduced stem cell frequency were observed in METTL3-inhibited SW620 and HCT116 cells compared with the corresponding control cells (Figs. 3a-b and Additional file 6: Figure S3a). The cell colony-formation and invasion abilities of SW620 and HCT116 cells were also impaired after METTL3 inhibition (Fig. 3c and Additional file 6: Figure S3b). As mentioned previously, stemness is thought to be responsible for chemotherapy resistance, and we specifically found that sensitivity to oxaliplatin-based chemotherapy was increased in METTL3-knockdown SW620 and HCT116 cells relative to the control cells (Fig. 3d and Additional file 6: Figure S3c). In addition, the expression of CSC surface antigens such as CD133, CD44, and epithelial cell adhesion molecule (EpCAM), in SW620 and HCT116 cells was remarkably reduced after METTL3 inhibition (Fig. 3e). Moreover, the expression of SOX2 downstream genes, including *cyclin D1 (CCND1), MYC proto-oncogene protein (MYC), and POU class 5 homeobox 1 (POU5F1)* [25–27], was consistently suppressed in METTL3-knockdown SW620 and HCT116 cells (Fig. 3f). These results revealed the oncogenic role of METTL3, specifically in the promoting of tumor self-renewal, cellular invasion and chemotherapy resistance in CRC cells.

We questioned whether SOX2 overexpression could rescue the reduction in stemness due to METTL3 inhibition. As expected, SOX2 overexpression in METTL3-knockdown and control CRC cells (Fig. 3g and Additional file 6: Figure S3d) led to the increased sphere formation, and an apparent chemotherapy resistance phenotype (Figs. 3h-i, and Additional file 6: Figures S3e-f). Collectively, the above results indicated the critical role of METTL3 in promoting stemness features through maintaining *SOX2* expression in CRC.

### METTL3 drives CRC tumorigenesis and metastasis in vivo

**Fig. 4:**
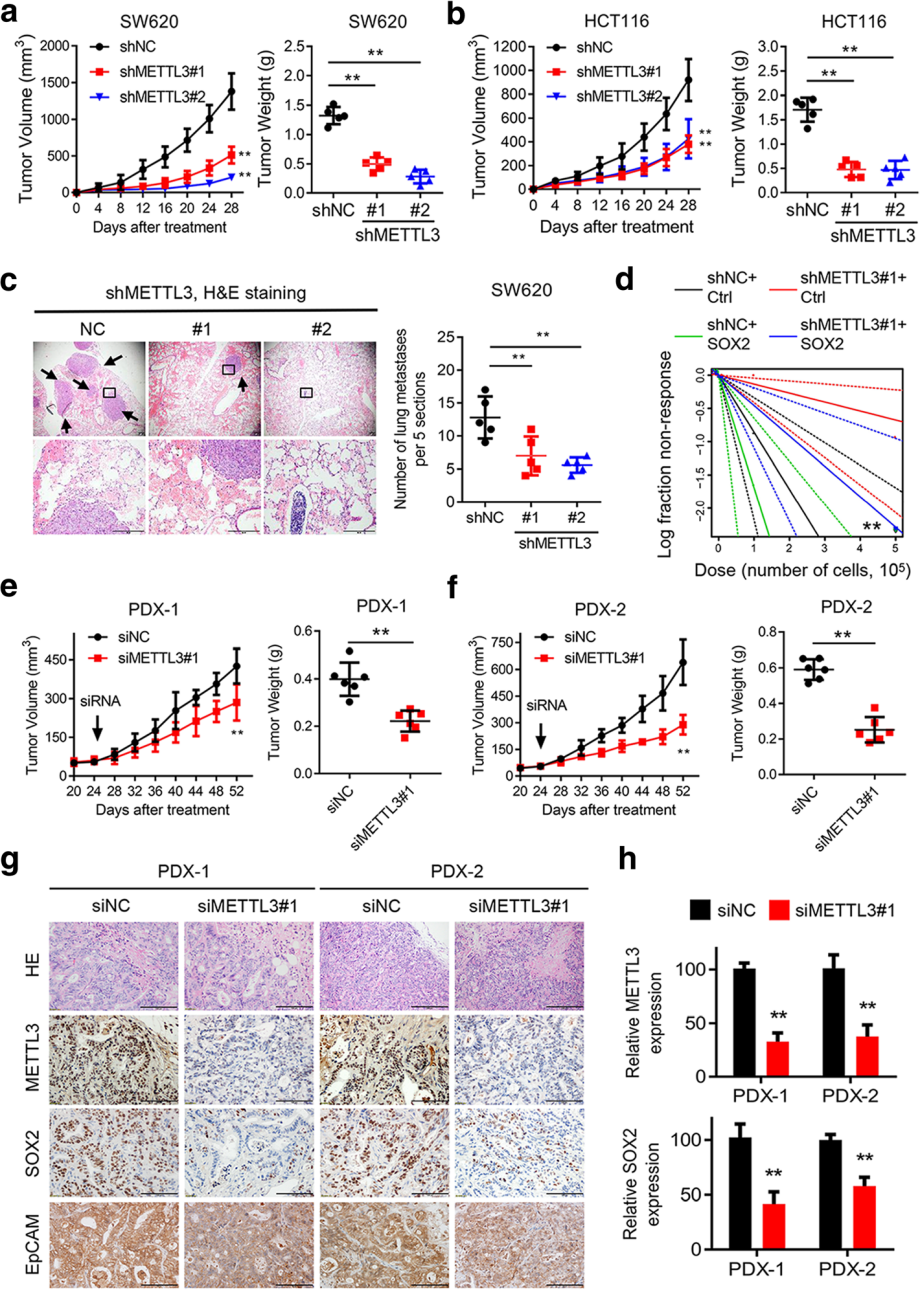
METTL3 drives CRC tumorigenesis and metastasis in vivo. **a-b,** Subcutaneous tumor models in nude mice showing the tumor growth rate (left) and tumor weights (right) at day 28 after the implantation of METTL3-knockdown and control SW620 and HCT116 cells (n = 5 mice per group). **c,** Representative H&E staining (scale bar: 100 μm) and quantification of metastatic lung nodules at day 60 after the tail vein injection of METTL3- knockdown or control SW620 cells (n = 5 mice per group). Arrow: metastatic lung nodules. Five sections were evaluated for each lung. **d,** In vivo limiting dilution assay showing the estimated frequency of CSCs among METTL3-knockdown and control SW620 cells with or without SOX2 overexpression. Response: mice developed subcutaneous tumor (n = 5 mice per group). **e-f,** Tumor growth rate and tumor weights in two PDX models of intratumoral treatment with siMETTL3 and siNC. **g-h,** Representative images and quantification of H&E and immunostaining (scale bar: 100 μm) of METTL3, SOX2, and EpCAM in two PDX-based subcutaneous tumor models. All data and error bars are presented as the mean ± SDs. *P < 0.05, **P < 0.01 (Student’s *t*-test)

To investigate the function of METTL3 in vivo, we next performed a subcutaneous xenotransplantation assay to determine whether METTL3 contributed to CRC development. The tumor growth rate was slower, and the xenograft tumor weight was reduced, when METTL3-knockdown SW620 and HCT116 cells were implanted, compared with the control cells (Figs. 4a-b, and Additional file 7: Figure S4a). Immunostaining assays indicated that the growth-impaired tumors generated from METTL3-ablated CRC cells had lower expression of SOX2 and EpCAM compared with the control subcutaneous mouse models (Additional file 7: Figure S4b). Moreover, compared with the mice that tail vein injected with METTL3 knockdown cells, the mice injected with control SW620 cells developed more lung metastatic nodules, as observed by histologic examination (Figs. 4c). As demonstrated above, METTL3 maintained self-renewal ability in vitro; therefore, we explored whether a similar effect would exist in vivo. Notably, the frequency of tumorigenic CRC cells was significantly decreased among the METTL3 knockdown SW620 cells (Fig. 4d, and Additional file 7: Figures S4c-d). In addition, SOX2 overexpression subsequently increased the tumor incidence and the frequency of tumorigenic cells among both control and METTL3 knockdown SW620 cells (Fig. 4d and Additional file 7: Figures S4c-d). Alltogether, the xenograft mouse models demonstrated that METTL3 contributed to tumorigenesis and the formation of metastatic foci through maintaining SOX2 expression in CRC.

PDX tumor models can simulate the physical tumor microenvironment, and the tumor growth corresponds to the treatment evaluations for the original patient [20, 28]. Therefore, we applied two PDX models to evaluate the potential therapeutic effect of METTL3 through intratumoral RNAi injection. In addition, the volumes of tumors treated with METTL3 siRNA were significantly lower than that of tumors in control group (Figs. 4e-f). Moreover, the PDX tumors were isolated and assessed by IHC staining, which showed reduced staining of METTL3, SOX2 and EpCAM expression in the METTL3 siRNA-treated group (Figs. 4g-h). Taken together, the above results further highlighted the crucial roles of METTL3 in CRC tumorigenesis and metastasis in vivo.

### IGF2BP2 enhances *SOX2* mRNA stability via an m^6^A-dependent manner

**Fig. 5:**
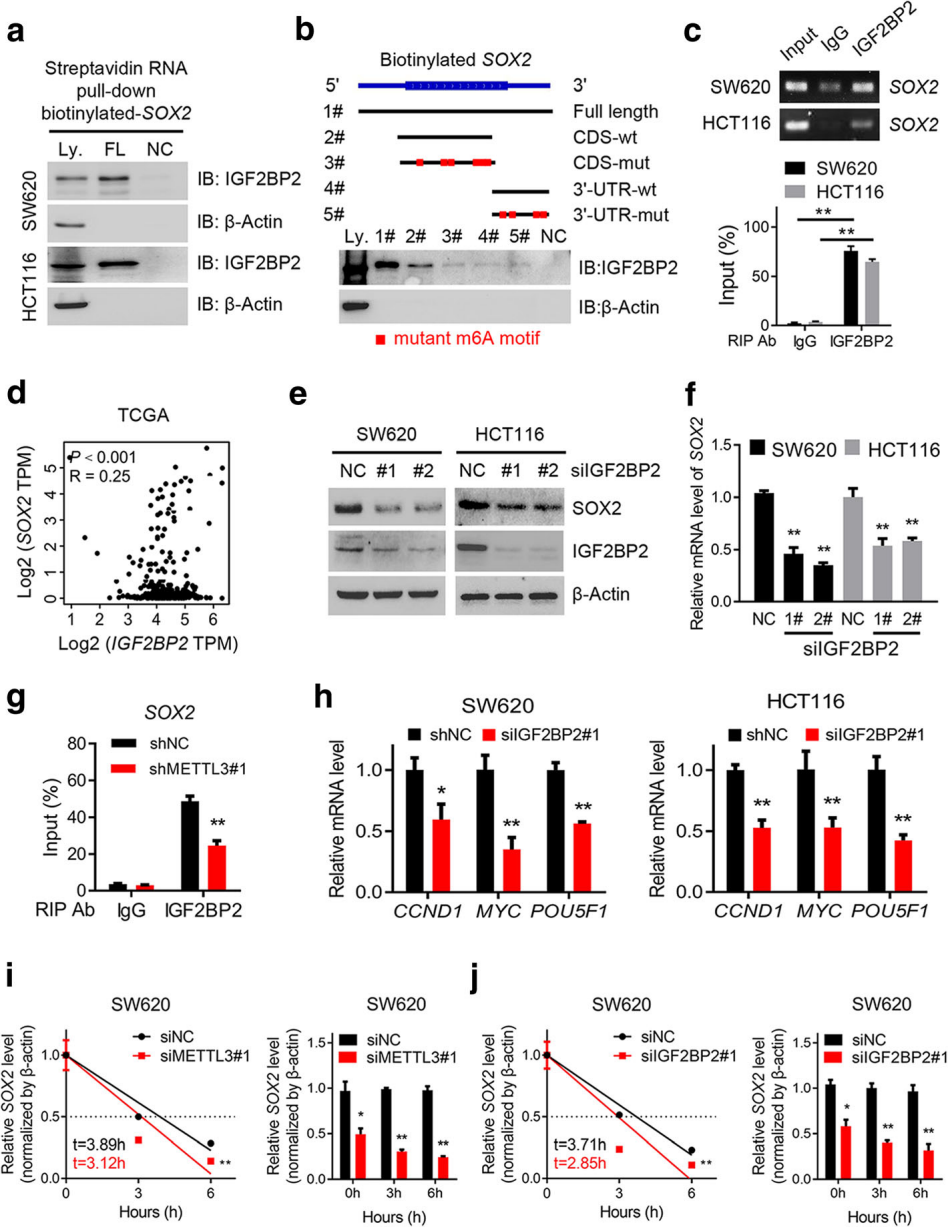
IGF2BP2 enhances *SOX2* mRNA stability via an m^6^A-dependent manner. **a,** Immunoblotting of IGF2BP2 after RNA pull down assay with cell lysate (Ly.), full-length biotinylated-*SOX2* (FL), and beads only (NC) in SW620 and HCT116 cells. **b,** Immunoblotting of IGF2BP2 with cell lysate (Ly.), full-length biotinylated-*SOX2* (#1), the *SOX2* CDS region with or without m^6^A motif mutation (#2, #3), the *SOX2* 3′-UTR region with or without m^6^A motif mutation (#4, #5), and beads only (NC) in SW620 cells. **c,** Agarose electrophoresis and real-time PCR analysis of RIP assays in CRC cells showing the direct binding between the IGF2BP2 protein and *SOX2* mRNA. **d,** Correlation between *IGF2BP2* and *SOX2* expression in TCGA database for COAD, analyzed with the Gene Expression Profiling Interactive Analysis (GEPIA) online analysis tool (http://gepia.cancer-pku.cn/). **e,** Immunoblotting of SOX2 after IGF2BP2 inhibition in SW620 and HCT116 cells. **f,** Real-time PCR analysis of *SOX2* after IGF2BP2 inhibition in SW620 and HCT116 cells. **g,** RIP-qPCR showing the enrichment of *SOX2* in SW620 after METTL3 inhibition. **h,** Real-time PCR analysis of SOX2 downstream genes after IGF2BP2 inhibition in SW620 and HCT116 cells. **i-j,** The decay rate of mRNA and qPCR analysis of SOX2 at the indicated times after actinomycin D (5 μg/ml) treatment in SW620 cells after METTL3 inhibition (left), and in SW620 cells after IGF2BP2 inhibition (right). The data in c, g, h, i and j are presented as the mean ± SDs (n = 3). *P < 0.05, **P < 0.01 (Student’s *t*-test). β-Actin and an IgG antibody was used as the negative control. The relative expression level was normalized by β-Actin. The relative *SOX2* enrichment in the RIP assay was normalized by input

Previous studies had identified two major families of m^6^A “readers” that might play a specific role in control the fate of the methylated mRNA, such as the YTH family and the IGF2BP family [12, 29, 30]. To elucidate the specific m^6^A readers of *SOX2,* and determine the m^6^A-dependent mechanism of *SOX2* regulation, we performed a streptavidin RNA pull-down assay to screen for *SOX2-*related m^6^A readers. Interestingly, IGF2BP2, but not other members of the IGF2BP family or the YTH family, specifically bound the *SOX2* full-length transcripts in SW620 and HCT116 cells (Fig. 5a and Additional file 8: Figure S5a). As represented in Fig. 2d, our m^6^A-seq data also provided a clue that the most of the m^6^A peaks of *SOX2* transcripts were located near the stop codon which indicated possible binding sites. Notably, the RNA pull-down assays verified that IGF2BP2 predominantly bound to the *SOX2* CDS region, instead of the 3′-UTR in SW620 cells, and the specific binding was significantly impaired after m^6^A motif depletion (Fig. 5b and Additional file 8: Figure S5b). RIP assays also validated the direct interaction between the IGF2BP2 and *SOX2* mRNA in SW620 and HCT116 cells (Fig. 5c). In TCGA database for COAD, a positive correlation between *IGF2BP2* and *SOX2* expression was observed as shown in Fig. 5d, indicating the potential positive regulatory mechanism. Consistent with our hypothesis, SOX2 protein and mRNA expression were significantly decreased after the siRNA inhibition of IGF2BP2 in SW620 and HCT116 cells (Figs. 5e-f). Additionally, the direct interaction between IGF2BP2 and *SOX2* transcripts was impaired in SW620 cells after METTL3 inhibition (Fig. 5g). The expression of SOX2 downstream genes, as shown in Fig. 3f, was also reduced after IGF2BP2 inhibition in SW620 and HCT116 cells (Fig. 5h). Furthermore, we assessed the RNA decay rate in METTL3 or IGF2BP2 inhibited CRC cells and the corresponding control cells. The *SOX2* mRNA expression was initially decreased and the *SOX2* mRNA half-lives were consistently markedly shortened upon METTL3 or IGF2BP2 inhibition in SW620 and HCT116 cells (Figs. 5i-j, and Additional file 8: Figures S5c-d). Taken together, our data suggested that the methylated *SOX2* transcripts were directly recognized by the m^6^A “reader”, IGF2BP2, which maintained the stability of the transcripts to prevent its degradation and naturally increase its expression via an m^6^A-IGF2BP2-dependent mechanism.

### Clinical correlation between METTL3, SOX2 and IGF2BP2 in CRC

**Fig. 6:**
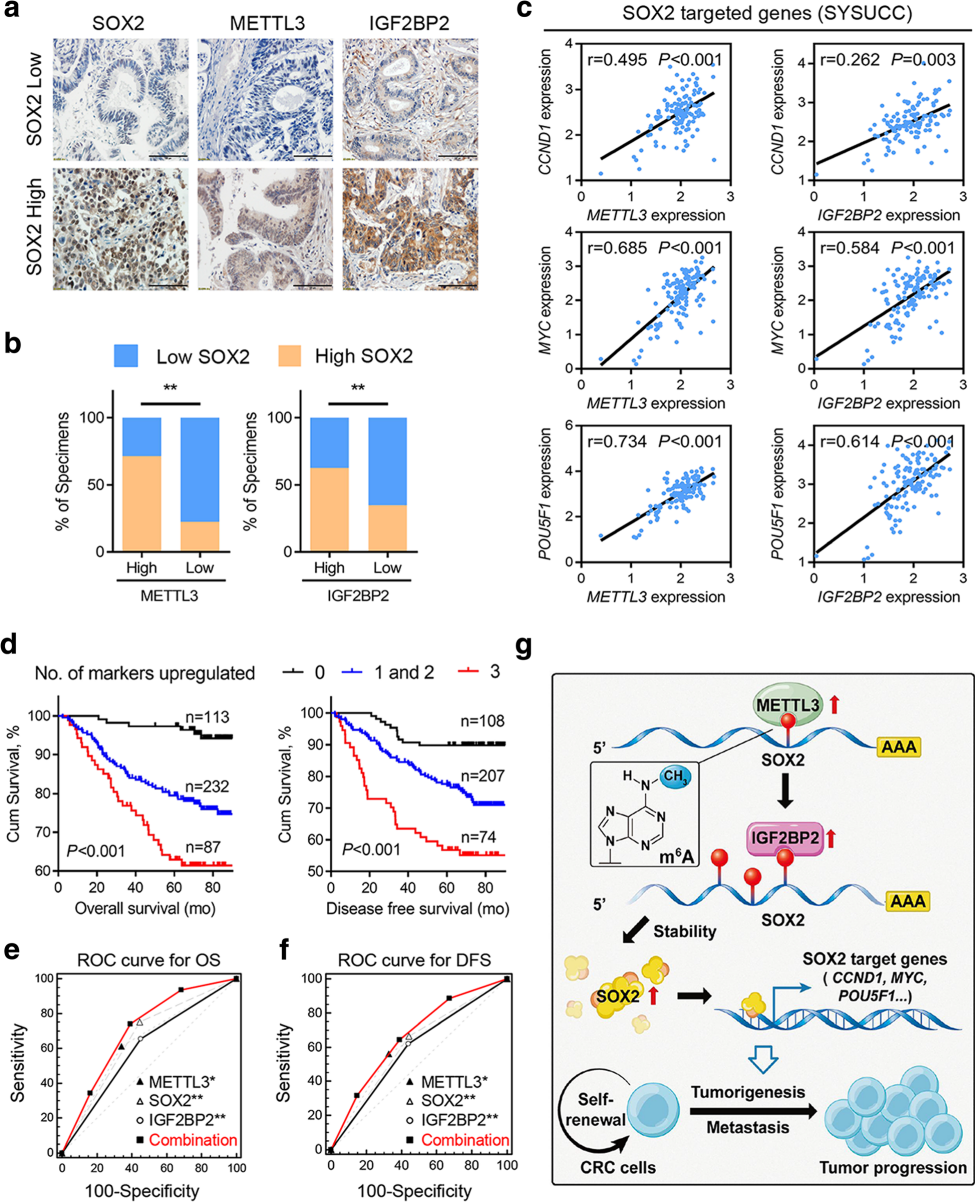
Clinical correlation between METTL3, SOX2 and IGF2BP2 in CRC. **a,** Representative images showing high or low expression of METTL3, SOX2 and IGF2BP2 in 432 CRC tumor specimens. **b,** Correlation between SOX2 and METTL3 or IGF2BP2 in CRC microarray specimens. **c,** Correlation between *METTL3* level (left) or *IGF2BP2* level (right) and the levels of SOX2 downstream genes, including *CCND1*, *MYC,* and *POU5F1*, in 63 paired CRC tumor tissues and adjacent normal tissues (SYSUCC cohort). **d,** Kaplan-Meier analysis of overall survival (OS) for CRC patients (*n* = 432) based on the number of upregulated molecular markers (Kaplan-Meier analysis with log-rank test). METTL3, SOX2, and IGF2BP2 expression was stratified by the individual medians by IHC analysis, and the patients were divided into three groups as indicated. **e,** ROC curve analysis for OS for METTL3 [AUC = 0.654, (95% CI, 0.607–0.698)], SOX2 [AUC = 0.635, (95% CI, 0.588–0.681)], and IGF2BP2 [AUC = 0.602, (95% CI, 0.554–0.649)] as individual biomarkers or for the combined panel [AUC = 0.703 (95% CI, 0.658–0.746)]. AUC, area under the curve. *P < 0.05, **P < 0.01 (Student’s *t*-test). **f,** ROC curve analysis for DFS for METTL3 [AUC = 0.612, (95% CI, 0.564–0.658)], SOX2 [AUC = 0.615, (95% CI, 0.568–0.661)], and IGF2BP2 [AUC = 0.591, (95% CI, 0.543–0.637)] as individual biomarkers or for the combined panel [AUC = 0.664 (95% CI, 0.618–0.709)]. AUC, area under a curve. **g,** Proposed working model of the proposed mechanism in this study. *P < 0.05, **P < 0.01 (Student’s *t*-test)

Based on the mechanism we identified above, we proceeded to explore the clinical relevance between METTL3, IGF2BP2, and SOX2 in our study. An IHC assay of SOX2, and IGF2BP2 was performed using the CRC microarray (Fig. 6a). IHC analysis showed that the expression of both SOX2 and IGF2BP2 was significantly increased in CRC tumor tissues compared with that in the paired adjacent normal tissues (Additional file 9: Figure S6a). Consistent with this finding, the Kaplan-Meier survival analysis and log-rank test suggested that high expression of SOX2 and IGF2BP2 notably correlated with shorter overall survival and disease-free survival times (Additional file 9: Figures S6b-c). Notably, SOX2 expression positively correlated with both METTL3 and IGF2BP2 in CRC tissues (Fig. 6b). Moreover, *METTL3* or *IGF2BP2* expression positively correlated with the SOX2 downstream genes *CCND1, MYC,* and *POU5F1* in our independent cohort of paired CRC tumor and adjacent normal tissues from SYSUCC (Fig. 6c). Similar results were also observed in TCGA database in a COAD cohort (Additional file 9: Figure S6d). Using Cox regression analysis, IHC scores for METTL3, SOX2, and IGF2BP2 expression were analyzed in a CRC patient cohort, and each of these three genes showed a notably increased hazard ratio (HR) for death, indicating that these three genes were independent prognostic factors in our CRC cohorts (Additional file 10: Table S3). Therefore, we attempted to generate a new IHC panel containing METTL3, SOX2, and IGF2BP2 to predict the prognosis of CRC. The Kaplan-Meier survival analysis and log-rank test suggested that the patients with three highly expressed markers had the shortest overall survival and disease-free survival times (Fig. 6d). Moreover, in the receiver operating characteristic (ROC) curve analysis, the combination index of the new IHC panel (METTL3, SOX2, and IGF2BP2) showed an additive predictive value for overall survival compared with any individual marker (Figs. 6e-f). The improved predictive values provided us with a credible IHC panel for evaluating the prognosis of CRC patients. As illustrated in Fig. 6g, METTL3 was highly expressed in CRC patients, and contributed to an increase in the m^6^A methylation level of *SOX2* transcripts. Methylated *SOX2* was subsequently recognized by the m^6^A “reader”, IGF2BP2, to maintain its mRNA stability and expression. Finally, increasing SOX2 expression promoted CRC cell stemness and metastasis through downstream targets of SOX2, leading to CRC progression.

## Discussion

METTL3, acting as the key component of N^6^-methyltransferase complex, has been reported to play an important role in many tumor types [12, 18, 19, 30–33]. Our results uncover a significant oncogenic role for METTL3 in tumor progression, though there are other studies that had suggested some controversial conclusions. Two independent previous studies stated that both METTL3 and FTO played an oncogenic role in acute myeloid leukemia through the diverse downstream targets [14, 33]. Two other studies stated that either increased or decreased METTL3 expression could respectively promote the self-renewal and tumorigenicity of glioma stem-like cells [32, 34]. Moreover, one study showed that high m^6^A modification promoted hepatocellular carcinoma progression which was ascribed to high METTL3 expression, while another study believed that was ascribed to low METTL14 expression [19, 35]. Considering the controversial conclusions of m^6^A and METTL3 in different cancer types, we believe that our current study has uncovered the underlying functions of WERs in CRC and showed the oncogenic role of METTL3 in promoting CRC stemness and metastasis, indicating the broad impact of METTL3 and m^6^A methylation on cancer development and precision therapy.

Colorectal CSCs are a group of tumor cells with self-renewal ability and multiple differentiation potentials which have strong tumorigenic and metastatic potential [3, 4]. In our previous work, the presence of CSCs in CRC was also suggested to be responsible for chemotherapy resistance [36]. Therefore, the elimination of colorectal CSCs is an important therapeutic strategy to improve the prognosis of CRC patients [37, 38]. Specifically, our study demonstrated that the inhibition of METTL3 could augment the chemotherapy response and decrease the stem cell frequency in CRC both in vitro and in vivo. Moreover, inhibition of METTL3 with siRNA treatment could significantly reduce the tumor size in PDX models. These results suggested that inhibition of METTL3 may be an effective way to diminish CSCs, thereby terminating malignant tumor recurrence and metastasis.

The acknowledged CSCs marker SOX2, was previously reported to be highly expressed and to participate in maintaining the properties of tumor-initiating cells, promoting proliferation in squamous cell carcinoma [23, 39]. CD133, CD166, EpCAM and CD44 are reported to be surface antigen of colorectal CSCs [38]. However, the regulatory mechanism for these CSC markers remains unclear. In this study, we found that inhibition of METTL3 could basically reduce these surface antigens expression, confirmed the oncogenic effect of SOX2 and revealed the m^6^A-dependent regulatory mechanism to partially explain the common upregulation of SOX2 in CRC. MYC, as one of the SOX2 target gene [27], is reported to be directly controlled by METTL3/IGF2BP2 axis [29]. In our work, we think that MYC can be regulated by both METTL3/IGF2BP2 axis and SOX2 respectively, which might partially explain the elevated expression of MYC in various human cancers. In conclusion, we suggested that METTL3 might be a new CSC marker due to its functions in maintaining the CSC stemness phenotype, providing new ideas and theoretical basis for the diagnosis and treatment of CRC.

M^6^A readers were reported to be involved in controlling the fate of mRNA, and both the YTHDF2 and IGF2BP1/2/3 were associated with methylated mRNA stability [29, 30]. Our data first identified that only IGF2BP2 directly bound to the specific m^6^A sites in *SOX2* CDS regions and controlled the *SOX2* mRNA half-life via an m^6^A-dependent manner. In fact, before its identification as an m^6^A reader, IGF2BP2 had already suggested to be associated with tumor progression through preserving the stemness phenotype in glioblastoma and hepatocellular carcinoma [40–42]. Here, we coincidentally verified the high expression of IGF2BP2 in CRC and its regulatory effect on *SOX2* mRNA stability to promote CRC stemness. These results might partially account for the roles of IGF2BP2 in preserving the tumor stemness phenotype. However, further molecular mechanisms underlying m^6^A methylation and mRNA fate deserves extensive study.

## Conclusions

In conclusion, our study suggested that METTL3 was essential for CRC progression and provided an attractive m^6^A-dependent regulatory mechanism. The combined network of “writer” METTL3, “reader” IGF2BP2, and “target” SOX2 highlighted an innovative m^6^A-dependent gene regulatory mechanism in epigenetics. In addition, the PDX models indicated a promising therapeutic strategy for CRC through the use of the efficient inhibitors of METTL3, which we will focus on developing in the future.

## Additional files

Additional file 1: **Table S1.** Correlation analysis for clinicopathologic variables in METTL3 expression among 432 colorectal cancer patients. (DOCX 15 kb)

Additional file 2: **Table S2.** The specific sequence of wide-type or m^6^A motif depletion *SOX2* CDS and 3′-UTR. (DOCX 14 kb)

Additional file 3: Supplementary materials and methods. (DOCX 35 kb)

Additional file 4: **Figure S1**, related to Fig. 1. METTL3 is highly expressed in human tumors. a, Real-time PCR analysis of m^6^A WER expression in 48 paired CRC tumor tissues (T) and adjacent normal tissues (N). b, Box plots of METTL3 expression in TCGA database. (TIF 6470 kb)

Additional file 5: **Figure S2**, related to Fig. 2: Identification of METTL3 targets via MeRIP-seq and RNA-seq. a, Volcano Plots showing the numbers of transcripts with significantly increased and decreased m^6^A peaks (fold change > 1.5 or < − 1.5, *P* < 0.05) in SW620 cells compared with SW480 cells (left) and in METTL3-knockdown SW620 compared with the control SW620 cells (right). b, Venn diagram showing the shared peaks between metastatic-related hyper-methylated peaks with METTL3-related hypo-methylated peaks. c, Gene-specific m^6^A qPCR analysis of alterations in the m^6^A level in four representative genes in METTL3 knockdown HCT116 compared with the control cells. d, Real-time PCR analysis of mRNA expression of four representative genes in METTL3 knockdown and control SW620 and HCT116. The data in c, and d are presented as the means ± SDs (*n* = 3). *P < 0.05, **P < 0.01 (Student’s *t*-test). The relative m^6^A level was normalized by input. The relative expression level was normalized by β-Actin. (TIF 6844 kb)

Additional file 6: **Figure S3**, related to Fig. 3: METTL3 promotes CRC cell stemness in vitro. a, In vitro limiting dilution assay of METTL3-knockdown and control HCT116. A well not containing spheres (diameter ≥ 50 μm) was defined as a non-response (*n* = 12). b, Total number of colonies formed by METTL3-knockdown versus control SW620 and HCT116 cells. c, Cell viability of METTL3-knockdown HCT116 versus control HCT116 cells after oxaliplatin treatment for 48 h. d, Immunoblotting analysis of SOX2 and METTL3 in METTL3 knockdown and control HCT116 cells with or without SOX2 overexpression. e, Quantification of the in vitro sphere-formation assay of METTL3-knockdown and control HCT116 cells with or without SOX2 overexpression (*n* = 6). f, Cell viability of METTL3-knockdown and control HCT116 cells with or without SOX2 overexpression when treated with oxaliplatin for 48 h. All data are presented as the mean ± SDs (n = 3). *P < 0.05, **P < 0.01 (Student’s *t*-test). β-Actin was used as the loading control. (TIF 6517 kb)

Additional file 7: **Figure S4**, related to Fig. 4: METTL3 drives CRC tumorigenesis and metastasis in vivo. a, Subcutaneous tumor models in nude mice showing the tumor size at day 28 after the implantation of METTL3-knockdown and control SW620 and HCT116 cells (*n* = 5 mice per group). b, Representative and quantification of H&E and immunostaining (scale bar: 100 μm) of METTL3, SOX2, and EpCAM in subcutaneous tumor models of METTL3 knockdown and control SW620 and HCT116 cells. c, Tumor incidence showing the tumorigenesis of the indicated serial of cell numbers of METTL3 knockdown and control SW620 cells with or without SOX2 overexpression. d, Stem cell frequencies of METTL3 knockdown and control SW620 cells with or without SOX2 overexpression. Estimate: 1/ (the estimated stem cell frequency); Lower, Upper: 95% confidence intervals. All data and error bars are presented as the mean ± SDs. *P < 0.05, **P < 0.01 (Student’s *t*-test). (TIF 9763 kb)

Additional file 8: **Figure S5** related to Fig. 5: IGF2BP2 enhances *SOX2* mRNA stability via an m^6^A-dependent manner. a, Immunoblotting of IGF2BP1, IGF2BP3, YTHDF1, YTHDF2 after RNA pull down assay with cell lysate (Ly.), full-length biotinylated-*SOX2* (FL), and beads only (NC) in SW620 and HCT116 cells. b, Immunoblotting of IGF2BP1, IGF2BP3, YTHDF1, and YTHDF2 with cell lysate (Ly.), full-length biotinylated-*SOX2* (#1), the *SOX2* CDS region with or without m^6^A motif mutation (#2, #3), the *SOX2* 3′-UTR region with or without m^6^A motif mutation (#4, #5), and beads only (NC) in SW620 cells. c-d, The decay rate of mRNA and qPCR analysis of SOX2 at indicated time after actinomycin D (5 μg/ml) treatment in HCT116 cells after METTL3 inhibition (left), and in HCT116 cells after IGF2BP2 inhibition (right). The date in c, and d are presented as the mean ± SDs (n = 3). *P < 0.05, **P < 0.01 (Student’s *t*-test). β-Actin was used as the negative control. The relative expression level was normalized by β-Actin. (TIF 7319 kb)

Additional file 9: **Figure S6** related to Fig. 6: Clinical correlation between METTL3, SOX2 and IGF2BP2 in CRC. a, SOX2 and IGF2BP2 IHC staining scores in primary CRC tumor tissues (T) and adjacent normal tissue (ANT) (*n* = 432). b-c, Kaplan-Meier analysis of OS and DFS curves based on the expression of SOX2 and IGF2BP2 expression (Kaplan-Meier analysis with the log-rank test). d, Correlation between METTL3 level (left) or IGF2BP2 level (right) with SOX2 target genes, including *CCND1*, *MYC,* and *POU5F1*, in TCGA database for COAD. *P < 0.05, **P < 0.01 (Student’s *t*-test). (TIF 6918 kb)

Additional file 10: **Table S3** Univariate and multivariate analyses of prognostic factors for overall survival among 432 colorectal cancer patients. (DOCX 13 kb)

## Data Availability

All data generated or analyzed during this study are included either in this article or in the additional files. The MeRIP-seq data and RNA-seq data have been deposited in the Genome Sequence Archive (http://gsa.big.ac.cn/) and are accessible under GSA: CRA001257.

## Abbreviations

3′-UTR: three prime untranslated region
BCHE: Butyrylcholinesterase
CCND1: Cyclin D1
CDS: Coding sequence
COAD: Colon adenocarcinoma
CRC: Colorectal carcinoma
CSCs: Cancer stem-like cells
EpCAM: Epithelial cell adhesion molecule
FTO: Fat-mass and obesity-associated protein
HR: Hazard ratio
IGF2BP1: Insulin like growth factor 2 mRNA binding protein 1
IGF2BP2: Insulin like growth factor 2 mRNA binding protein 2
IHC: Immunohistochemical
m^6^A: N^6^-methyladenosine
MeRIP-seq: Methylated RNA immunoprecipitation sequencing
METTL3: Methyltransferase-like 3
MYC: MYC proto-oncogene protein
PDX: Patient-derived xenograft
POU5F1: POU class 5 homeobox 1
RIP: RNA immunoprecipitation
RNA-seq: transcriptomic RNA sequencing
ROC: Receiver operating characteristic
RT-qPCR: quantitative real-time PCR
SEMA3A: Semaphorin 3A
SOX2: SRY (sex determining region Y)-box 2
SYSUCC: Sun Yat-sen University Cancer Center
TCGA: The Cancer Genome Atlas
YTHDF1: YTH N6-methyladenosine RNA binding protein 1
YTHDF2: YTH N6-methyladenosine RNA binding protein 2
ZFP36L2: ZFP36 ring finger protein like 2
