# Supplementary material for: METTL3 facilitates tumor progression via an m6A-IGF2BP2-dependent mechanism in colorectal carcinoma
Source: Mol Cancer. 2019 Jun 24;18:112. doi: 10.1186/s12943-019-1038-7 (PMC6589893; doi:10.1186/s12943-019-1038-7)
Supplement: Supplementary file 1 — Table S1. Correlation analysis for clinicopathologic variables in METTL3 expression among 432 colorectal cancer patients. (DOCX 15 kb) [file 12943_2019_1038_MOESM1_ESM.docx]

**Table S1: Correlation analysis for clinicopathologic variables in METTL3 expression among 432 colorectal cancer patients**

| Variable | low METTL3  n (%) | high METTL3  n (%) | *P* value |
| --- | --- | --- | --- |
| Total | 211 (48.8) | 221 (51.2) |  |
| Age, years |  |  | 0.488 |
| ≤ 60 | 127 (29.4) | 268 (32.6) |  |
| > 60 | 84 (19.4) | 80 (18.5) |  |
| Gender |  |  | 0.283 |
| Male | 120 (27.8) | 137 (31.7) |  |
| Female | 91 (31.7) | 84 (19.4) |  |
| Clinical stage |  |  | 0.203 |
| Ⅰ-Ⅱ | 77 (17.8) | 94 (21.8) |  |
| Ⅲ-Ⅳ | 134 (31.0) | 127 (29.4) |  |
| Degree of differentiation |  |  | 0.163 |
| Well/moderate | 171 (39.6) | 166 (38.4) |  |
| Poor | 40 (9.3) | 55 (12.7) |  |
| Tumor depth |  |  | 0.330 |
| m/sm/mp | 24 (5.6) | 18 (4.2) |  |
| ss/se/si | 187 (43.3) | 203 (47.0) |  |
| Vascular invasion |  |  | 0.245 |
| Absent | 170 (39.4) | 167 (38.7) |  |
| Present | 41 (9.5) | 54 (12.5) |  |
| Perineural invasion |  |  | 0.441 |
| Absent | 117 (27.1) | 114 (26.4) |  |
| Present | 94 (21.8) | 107 (24.8) |  |
| Abbreviations: m: tumor invasion of mucosa; sm: submocosa; mp: muscular is propria; ss:subserose; se: serosa penetration; si: invasion to adjacent structures. | | | |
