# Supplementary material for: METTL3 facilitates tumor progression via an m6A-IGF2BP2-dependent mechanism in colorectal carcinoma
Source: Mol Cancer. 2019 Jun 24;18:112. doi: 10.1186/s12943-019-1038-7 (PMC6589893; doi:10.1186/s12943-019-1038-7)
Supplement: Supplementary file 2 — Table S2. The specific sequence of wide-type or m6A motif depletion SOX2 CDS and 3′-UTR. (DOCX 14 kb) [file 12943_2019_1038_MOESM2_ESM.docx]

**Table S2: Univariate and multivariate analyses of prognostic factors for overall survival among 432 colorectal cancer patients.**

| Factors | Univariate | | Multivariate* | |
| --- | --- | --- | --- | --- |
|  | HR (95% CI) | *P* value | HR (95% CI) | *P* value |
| Age  (<=60/>60) | 0.565 (0.361-0.884) | 0.012 | 2.136 (1.414-3.228) | 0.000 |
| Gender  (male/female) | 1.824 (1.214-2.740) | 0.004 | / | / |
| TNM stage  (Ⅰ-Ⅱ/Ⅲ-Ⅳ) | 3.443 (2.033-5.829) | 0.000 | 3.094 (1.735-5.519) | 0.000 |
| Histological grade  (well, moderate/poor) | 1.563 (0.999-2.446) | 0.051 | / | / |
| Tumor depth  (m, sm, mp/ss, se, si) | 1.705 (0.745-3.900) | 0.206 | / | / |
| Vascular invasion  (absent/present) | 3.929 (2.607-5.921) | 0.000 | 1.685 (1.031-2.745) | 0.037 |
| Perineural invasion  (absent/present) | 3.193 (2.049-4.977) | 0.000 | 2.088 (1.269-3.435) | 0.004 |
| METTL3 expression  (low/high) | 3.750 (2.316-6.072) | 0.000 | 3.259 (1.919-5.536) | 0.000 |
| SOX2 expression (low/high) | 2.904 (1.905-4.427) | 0.000 | 1.694 (1.042-2.756) | 0.034 |
| IGF2BP2 expression (low/high) | 2.175 (1.416-3.343) | 0.000 | 1.713 (1.101-2.666) | 0.017 |
| Abbreviations: CRC: Colorectal cancer; m: tumor invasion of mucosa; sm: submocosa; mp: muscular is propria; ss:subserose; se: serosa penetration; si: invasion to adjacent structures. HR: hazard ratio; CI: confidence interval.  * For the multivariate model, HR and *P* values were shown by backward method only. | | | | |
