# Supplementary material for: METTL3 facilitates tumor progression via an m6A-IGF2BP2-dependent mechanism in colorectal carcinoma
Source: Mol Cancer. 2019 Jun 24;18:112. doi: 10.1186/s12943-019-1038-7 (PMC6589893; doi:10.1186/s12943-019-1038-7)
Supplement: Supplementary file 3 — Supplementary materials and methods. (DOCX 35 kb) [file 12943_2019_1038_MOESM3_ESM.docx]

**Supplement Methods**

**Cells and cell culture**

The human CRC cell lines and immortalized colon epithelial cells (CCD112, CCD841) were obtained from the American Type Culture Collection (Manassas, VA, USA) and cultured by the manufacturer. All cells were tested negatively for mycoplasma contamination before use, and authenticated by STR fingerprinting before use at Medicine Lab of Forensic Medicine Department of Sun Yat-sen University.

**Immunoblotting and IHC analysis**

Immunoblotting and IHC analysis were conducted as previously reported[1, 2]. The blotting membranes were stripped and re-incubated with anti-β-Actin antibody for loading control. The IHC stained sections were reviewed and scored independently by two superior pathologists. A final score was then calculated by multiplying the score of proportion of positively stained tumor cells (0-100%) and the score of staining intensity (0,1,2,3).

The antibodies used for IHC and immunoblotting assay were listed as following: METTL3 (#ab195352), CD133 (#ab19898), CD44 (#ab51037), EpCAM (#ab32392), SOX2 (#ab92494), IGF2BP2 (ab#128175) (Abcam), IGF2BP1 (22803-1-AP), IGF2BP3 (14642-1-AP), YTHDF1 (17479-1-AP), YTHDF2 (24744-1-AP) (Proteintech), Flag (F1804), β-Actin (A5441) (Sigma).

**Lentiviral Transduction for Stable Cell Lines**

The small interfering RNA (siRNA) targeting METTL3 (targeting sequences: #1: GCACTTGGATCTACGGAAT, #2: CGACTACAGTAGCTGCCTT) and IGF2BP2 (targeting sequences: #1: CATGCCGCATGATTCTTGA, #2: GAACGAACTGCAGAACTTA) were synthesized by RiboBio (Guangzhou, China). Transfection experiments were performed with Lipofectamine 3000 (Invitrogen). The lentiviruses packaging METTL3 shRNA were purchased from GenePharma (Shanghai, China). To establish stable METTL3 knockdown cell lines, CRC cells were transduced by lentiviruses and selected with puromycin (2 µg/ml, S7417, Selleck) after 3 days production. `For the SOX2 rescue experiment, SOX2-CDS were cloned to pcDNA3.1 vector with FLAG tag, and then the empty vector or the SOX2-CDS were transduced to the METTL3 knockdown and scramble CRC cells, respectively.

**RNA isolation and qPCR analysis**

Total RNA was isolated with TRIzol reagent and cDNA was then synthesis with Prime Script RT Master Mix Kit (RR036A, Takara) and served as template for real-time PCR using an GoTaq qPCR Master Mix (A6001, Promega) according to the manufacturer’s instructions. All data were analysis and normalized to β-Actin.

**RT-PCR primers used:**

METTL3 (forward: 5’--3’: TTGTCTCCAACCTTCCGTAGT, reverse: 5’--3’: CCAGATCAGAGAGGTGGTGTAG.)

METTL14 (forward: 5’--3’: GAGTGTGTTTACGAAAATGGGGT, reverse: 5’--3’: CCGTCTGTGCTACGCTTCA.)

WTAP (forward: 5’--3’: ACTGGCCTAAGAGAGTCTGAAG, reverse: 5’--3’: GTTGCTAGTCGCATTACAAGGA.)

ALKBH5 (forward: 5’--3’: CGGCGAAGGCTACACTTACG, reverse: 5’--3’: CCACCAGCTTTTGGATCACCA.)

FTO (forward: 5’--3’: GCTGCTTATTTCGGGACCTG, reverse: 5’--3’: AGCCTGGATTACCAATGAGGA.)

YTHDF1 (forward: 5’--3’: ACCTGTCCAGCTATTACCCG, reverse: 5’--3’: TGGTGAGGTATGGAATCGGAG.)

YTHDF2 (forward: 5’--3’: CCTTAGGTGGAGCCATGATTG, reverse: 5’--3’: TCTGTGCTACCCAACTTCAGT.)

YTHDF3 (forward: 5’--3’: GGTGTATTTAGTCAACCTGGGG, reverse: 5’--3’: AAGAGAACTAGGTGGATAGCCAT.)

IGF2BP1 (forward: 5’--3’: GCTCTTTGGGGACAGGAAGC, reverse: 5’--3’: GGAGCTCACCTCTTCATCCG.)

IGF2BP2 (forward: 5’--3’: AGCTAAGCGGGCATCAGTTTG, reverse: 5’--3’: CCGCAGCGGGAAATCAATCT.)

IGF2BP3 (forward: 5’--3’: TATATCGGAAACCTCAGCGAGA, reverse: 5’--3’: GGACCGAGTGCTCAACTTCT.)

SOX2 (forward: 5’--3’: ATCAGGAGTTGTCAAGGCAGAG, reverse: 5’--3’: AGAGGCAAACTGGAATCAGGA.)

CCND1 (forward: 5’--3’: GCTGCGAAGTGGAAACCATC, reverse: 5’--3’: CCTCCTTCTGCACACATTTGAA.)

MYC (forward: 5’--3’: GGACGACGACGAGACCTTCATCAA, reverse: 5’--3’: CCAGCTTCTCTGAGACGAGCTT.)

POU5F1 (forward: 5’--3’: ACATGTGTAAGCTGCGGCC, reverse: 5’--3’: GTTGTGCATAGTCGCTGCTTG.)

BCHE (forward: 5’--3’: TGGCTCGGGTTGAAAGAGTTA, reverse: 5’--3’: TCCTGGCAAAGCTAAGAATCCT.)

SEMA3A (forward: 5’--3’: GTGCCAAGGCTGAAATTATCCT, reverse: 5’--3’: CCCACTTGCATTCATCTCTTCT.)

ZFP36L2 (forward: 5’--3’: CAACTCCACGCGCTACAAGA, reverse: 5’--3’: CACTTTTCGCCGTACTTGCAC.)

β-Actin (forward: 5’--3’: TGGATCAGCAAGCAGGAGTA, reverse: 5’--3’: TCGGCCACATTGTGAACTTT.)

**Sequencing data analysis**

All samples from the MeRIP-seq libraries were performed on Illumina HiSeq 2500 or Illumina HiSeq X-10 with a single-end 50-bp read length aligned to the hg38 reference genome using STAR[3]. For the MeRIP-seq analysis: MACS2 was used to call m^6^A peaks based on the RNA-seq and m^6^A-seq BAM files from our input (RNA-seq) and m^6^A IP (MeRIP-seq) sequencing libraries. To achieve high specificity, only the m^6^A peaks called by MACS2 and identified in at least two samples were retained for the subsequent analysis. The m^6^A peaks were annotated using our custom Perl script and the m^6^A reads were extracted using BEDTools[4], and converted to RPKM using our custom Perl script. The differentially methylated m^6^A peaks were identified by using the “limma” package in R software[5]. For the RNA-seq analysis: All samples from the RNA-seq libraries were performed on Illumina HiSeq 2500 or Illumina HiSeq X-10 with a single-end 150-bp read length aligned to the hg38 reference genome using STAR[3]. The RNA-seq reads were normalized using the RSEM method[6] and “edgeR”[7] to find differentially expressed genes between two groups. For the gene enrichment analysis: The GO annotation enrichment analysis was performed on the list of the 158 shared genes by using a publicly online tool (<http://metascape.org/gp/index.html>). For data accession: all raw data and processed files have been deposited in the Genome Sequence Archive (<http://gsa.big.ac.cn/>) and are accessible under GSA: CRA001257.

**Cell invasion assay**

The standard procedure was performed as previously described[8]. Briefly, stable SW620 and HCT116 METTL3 knockdown or control cells were harvested and suspended in 200 μl serum-free medium (2×10^5^), and transferred to the upper chamber with the Matrigel-coated membrane (24-well insert; 8 μm pore, BD Biosciences), the lower chambers were filled with serum as a chemo-attractant. Invading cells were fixed, stained and counted in 5 random view fields after 24 hours.

**Cell sphere formation and colony formation assays**

2×10^3^ cells were plated in 96-well ultralow attachment plates (7007, Corning) with serum-free DMEM-F12 medium with 20 ng/ml epidermal growth factor (EGF) (100-47, Peprotech), 20 ng/ml of basic fibroblast growth factor (bFGF) (100-18B, Peprotech), 10 μg/ml heparin (H3149, Sigma), 2% B27 (17504044, Gibco). Then, diameter of the spheres greater than 50 μm in each well were counted under a microscope after 7 days. For the colony formation assay, the cells were plated in 6-well plates for 500 cells and cultured for 2 weeks. The colonies were calculated after fixed and stained with 1% crystal violet.

***In vitro* limiting dilution assay**

The stable METTL3 knockdown and control CRC cells were harvested and the single-cell suspension were seeded at 3, 6, 12, 25, 50, and 100 cells per well into 96-well plates. The number of wells with spheres (diameter >=50 μm) for each group were counted at 10 days after seeding cells.

**Cell viability assay**

CRC cells (5×10^3^ cell/well) were seeded in a 96-well plates overnight and then exposed to oxaliplatin (1 μM, 2 μM, 5 μM, 10 μM. 20 μM), cell viability was determined using MTS assay as previous described[9].

***In vivo* tumorigenesis, metastasis, limiting dilution assay**

All animal experiments were performed in accordance with a protocol approved by our Institutional Animal Care. To evaluate the tumorigenic effect of METTL3, female BABL/c nude mice (4 to 5 weeks old) were injected subcutaneously with 2×10^6^ METTL3 knockdown or control CRC cells. Tumor volume was estimated every four days using the standard formula: 0.5×length×width^2^, where length is the longest diameter and width is the shortest diameter of the tumor. At day of 28 after implantation, the tumors were excised, imaged and weighed.

For the lung metastasis model, 2×10^6^ METTL3 knockdown or control SW620 cells were directly injected into the tail veins of nude mice. Sixty days later, all mice were sacrificed and the lungs were excised, imaged and paraffin embedded. Consecutive sections were generated and subjected to H&E staining. The number of micro-metastatic nodules for each group was evaluated from 5 random areas using dissecting microscopes.

To investigate the effect of METTL3 and SOX2 on tumor self-renewal, an *in vivo* limiting dilution assay was performed. Female BABL/c nude mice (4 to 5 weeks old) were randomly divided into 4 groups (5 mice per group) randomly. METTL3-knockdown or control SW620 cells with or without SOX2 overexpression were injected subcutaneously in the right flank with a serial dilution of cells (1×10^3^, 1×10^4^, 1×10^5^, 5×10^5^). At the end of 45 days, the tumor incidence of each group was determined and the stem cell frequency was estimated using an online tool at <http://bioinf.wehi.edu.au/software/elda.>

**Patient-derived xenograft (PDX) models and intra-tumoral injection therapy**

PDX tumors were initially generated using fresh tumor tissues from two CRC patients that were implanted into F0 mice. When an appropriate-sized tumor was generated, the tumors were dissected, divided into equals volume and subcutaneously implanted into BABL/c nude mice (6 mice per group). 24 days later, cholesterol-modified METTL3 siRNA or control siRNA (RiboBio, 5 nmol) was injected precisely into the center of the xenografted tumors every 4 days. The mice were sacrificed, and the tumors were excised, imaged and weighed after 8 treatments. All animal studies were approved by the Institutional Animal Care and Use Committee at our institution.

**Reference**

1. Ju HQ, Lu YX, Chen DL, Tian T, Mo HY, Wei XL, Liao JW, Wang F, Zeng ZL, Pelicano H, et al: **Redox Regulation of Stem-like Cells Though the CD44v-xCT Axis in Colorectal Cancer: Mechanisms and Therapeutic Implications.** *Theranostics* 2016, **6:**1160-1175.

2. Lu YX, Ju HQ, Liu ZX, Chen DL, Wang Y, Zhao Q, Wu QN, Zeng ZL, Qiu HB, Hu PS, et al: **ME1 Regulates NADPH Homeostasis to Promote Gastric Cancer Growth and Metastasis.** *Cancer Res* 2018, **78:**1972-1985.

3. Dobin A, Davis CA, Schlesinger F, Drenkow J, Zaleski C, Jha S, Batut P, Chaisson M, Gingeras TR: **STAR: ultrafast universal RNA-seq aligner.** *Bioinformatics* 2013, **29:**15-21.

4. Quinlan AR: **BEDTools: The Swiss-Army Tool for Genome Feature Analysis.** *Curr Protoc Bioinformatics* 2014, **47:**11.12.11-34.

5. Ritchie ME, Phipson B, Wu D, Hu Y, Law CW, Shi W, Smyth GK: **limma powers differential expression analyses for RNA-sequencing and microarray studies.** *Nucleic Acids Res* 2015, **43:**e47.

6. Li B, Dewey CN: **RSEM: accurate transcript quantification from RNA-Seq data with or without a reference genome.** *BMC Bioinformatics* 2011, **12:**323.

7. Robinson MD, McCarthy DJ, Smyth GK: **edgeR: a Bioconductor package for differential expression analysis of digital gene expression data.** *Bioinformatics* 2010, **26:**139-140.

8. Chen DL, Lu YX, Zhang JX, Wei XL, Wang F, Zeng ZL, Pan ZZ, Yuan YF, Wang FH, Pelicano H, et al: **Long non-coding RNA UICLM promotes colorectal cancer liver metastasis by acting as a ceRNA for microRNA-215 to regulate ZEB2 expression.** *Theranostics* 2017, **7:**4836-4849.

9. Ju HQ, Lu YX, Wu QN, Liu J, Zeng ZL, Mo HY, Chen Y, Tian T, Wang Y, Kang TB, et al: **Disrupting G6PD-mediated Redox homeostasis enhances chemosensitivity in colorectal cancer.** *Oncogene* 2017, **36:**6282-6292.
