# Supplementary figures and images for: METTL3 facilitates tumor progression via an m6A-IGF2BP2-dependent mechanism in colorectal carcinoma
Source: Mol Cancer. 2019 Jun 24;18:112. doi: 10.1186/s12943-019-1038-7 (PMC6589893; doi:10.1186/s12943-019-1038-7)

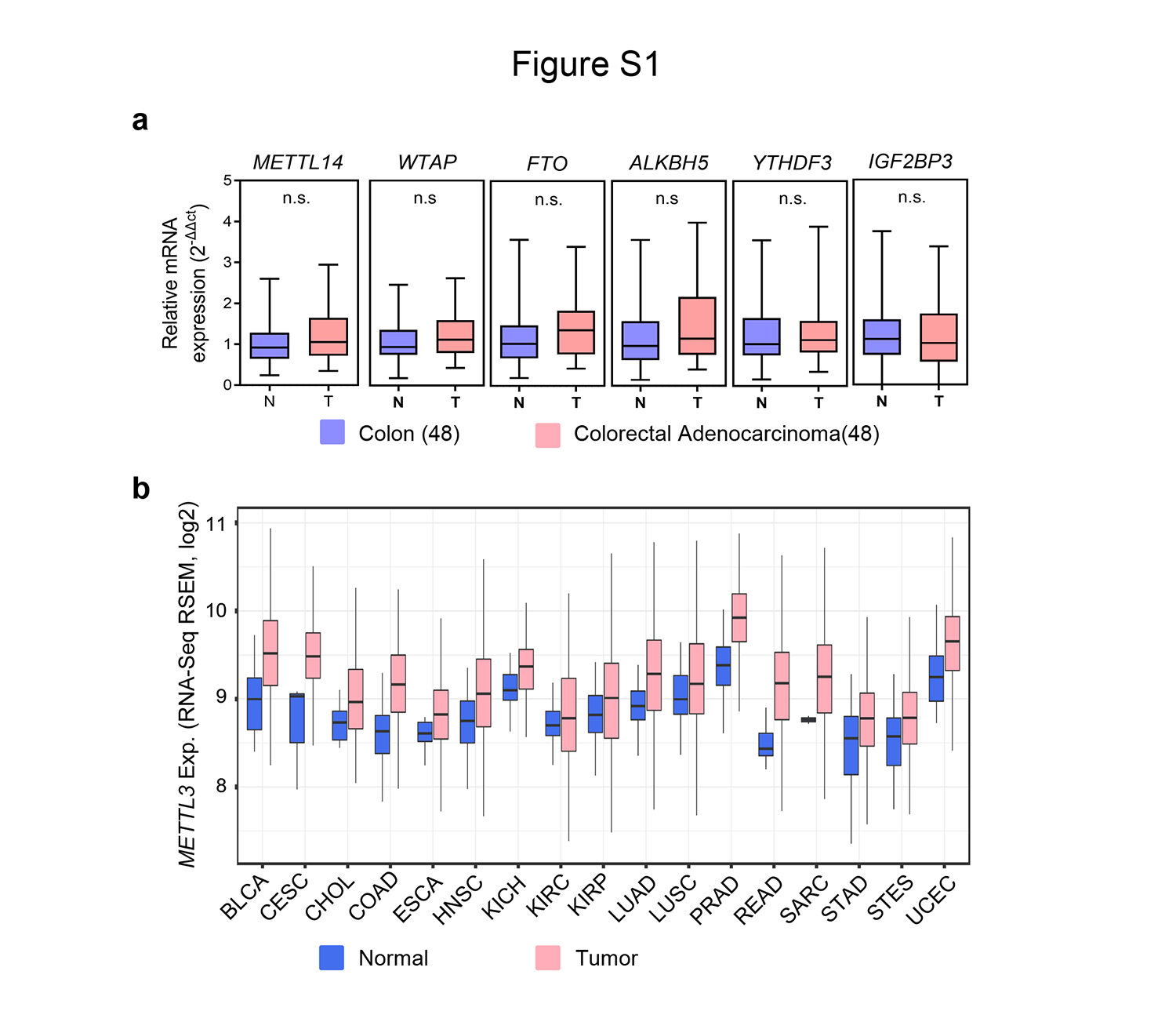

Supplement: Supplementary file 4 — Figure S1, related to Fig. 1. METTL3 is highly expressed in human tumors. a, Real-time PCR analysis of m6A WER expression in 48 paired CRC tumor tissues (T) and adjacent normal tissues (N). b, Box plots of METTL3 expression in TCGA database. (TIF 6470 kb) [file 12943_2019_1038_MOESM4_ESM.tif]

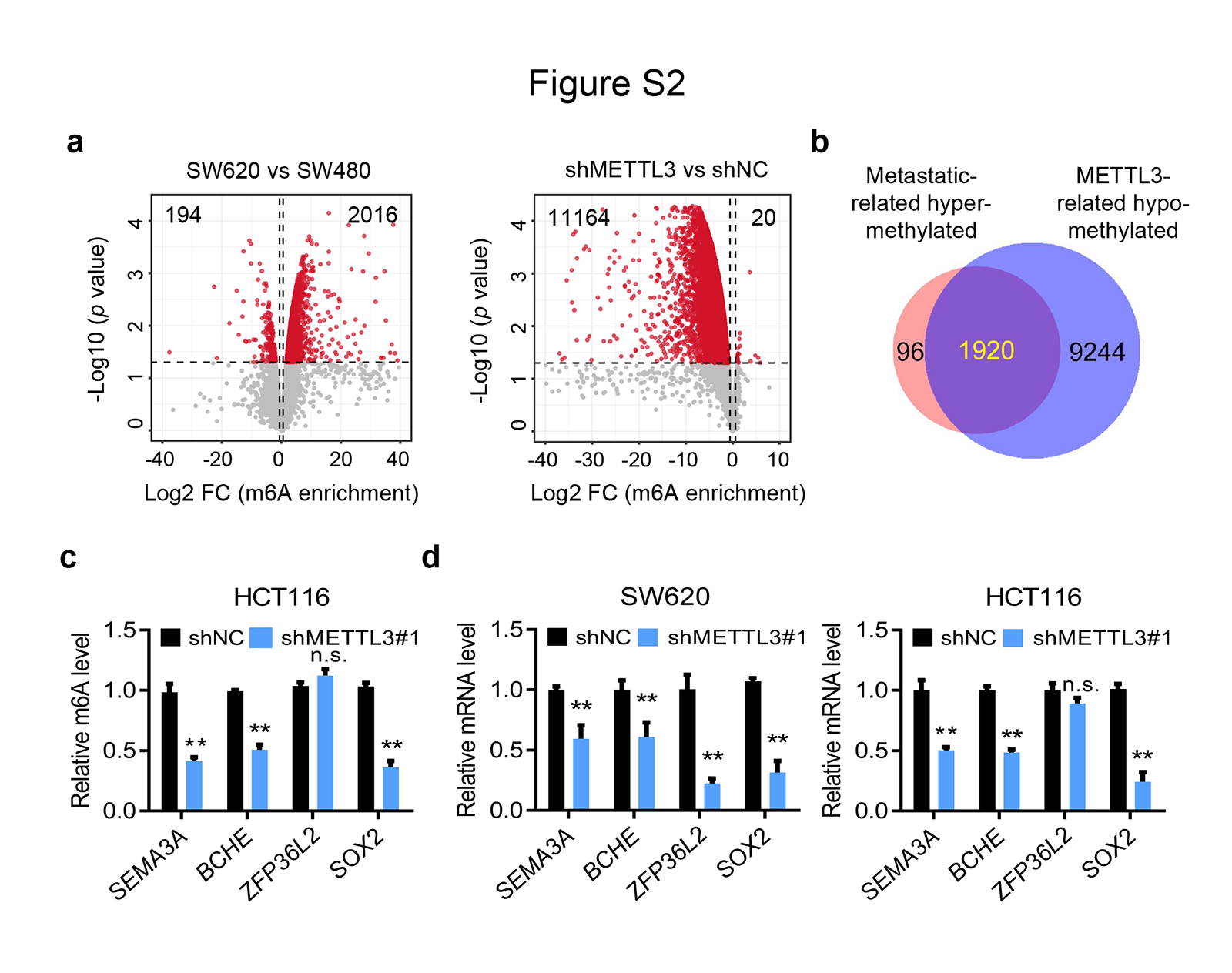

Supplement: Supplementary file 5 — Figure S2, related to Fig. 2: Identification of METTL3 targets via MeRIP-seq and RNA-seq. a, Volcano Plots showing the numbers of transcripts with significantly increased and decreased m6A peaks (fold change > 1.5 or < − 1.5, P < 0.05) in SW620 cells compared with SW480 cells (left) and in METTL3-knockdown SW620 compared with the control SW620 cells (right). b, Venn diagram showing the shared peaks between metastatic-related hyper-methylated peaks with METTL3-related hypo-methylated peaks. c, Gene-specific m6A qPCR analysis of alterations in the m6A level in four representative genes in METTL3 knockdown HCT116 compared with the control cells. d, Real-time PCR analysis of mRNA expression of four representative genes in METTL3 knockdown and control SW620 and HCT116. The data in c, and d are presented as the means ± SDs (n = 3). *P < 0.05, **P < 0.01 (Student’s t-test). The relative m6A level was normalized by input. The relative expression level was normalized by β-Actin. (TIF 6844 kb) [file 12943_2019_1038_MOESM5_ESM.tif]

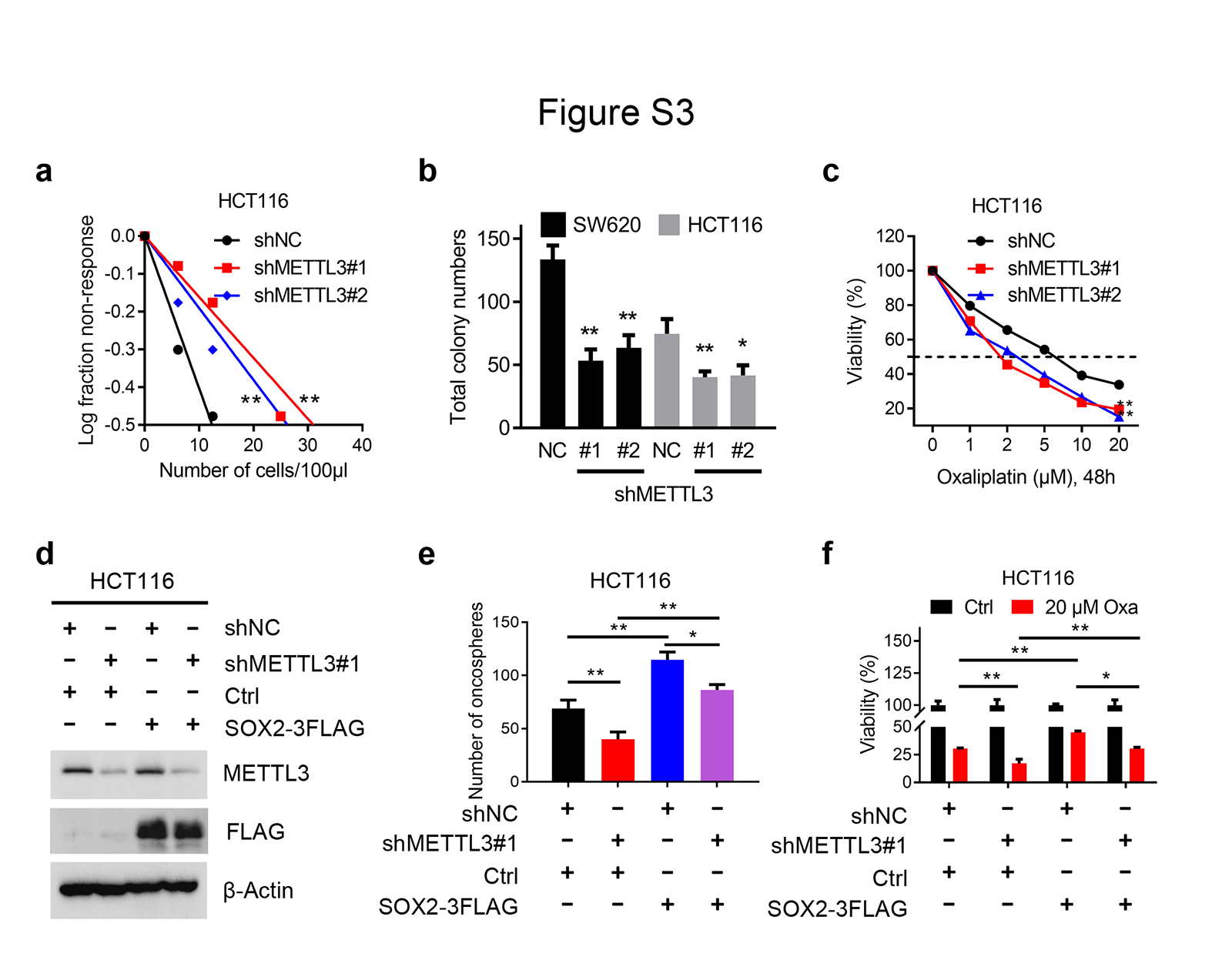

Supplement: Supplementary file 6 — Figure S3, related to Fig. 3: METTL3 promotes CRC cell stemness in vitro. a, In vitro limiting dilution assay of METTL3-knockdown and control HCT116. A well not containing spheres (diameter ≥ 50 μm) was defined as a non-response (n = 12). b, Total number of colonies formed by METTL3-knockdown versus control SW620 and HCT116 cells. c, Cell viability of METTL3-knockdown HCT116 versus control HCT116 cells after oxaliplatin treatment for 48 h. d, Immunoblotting analysis of SOX2 and METTL3 in METTL3 knockdown and control HCT116 cells with or without SOX2 overexpression. e, Quantification of the in vitro sphere-formation assay of METTL3-knockdown and control HCT116 cells with or without SOX2 overexpression (n = 6). f, Cell viability of METTL3-knockdown and control HCT116 cells with or without SOX2 overexpression when treated with oxaliplatin for 48 h. All data are presented as the mean ± SDs (n = 3). *P < 0.05, **P < 0.01 (Student’s t-test). β-Actin was used as the loading control. (TIF 6517 kb) [file 12943_2019_1038_MOESM6_ESM.tif]

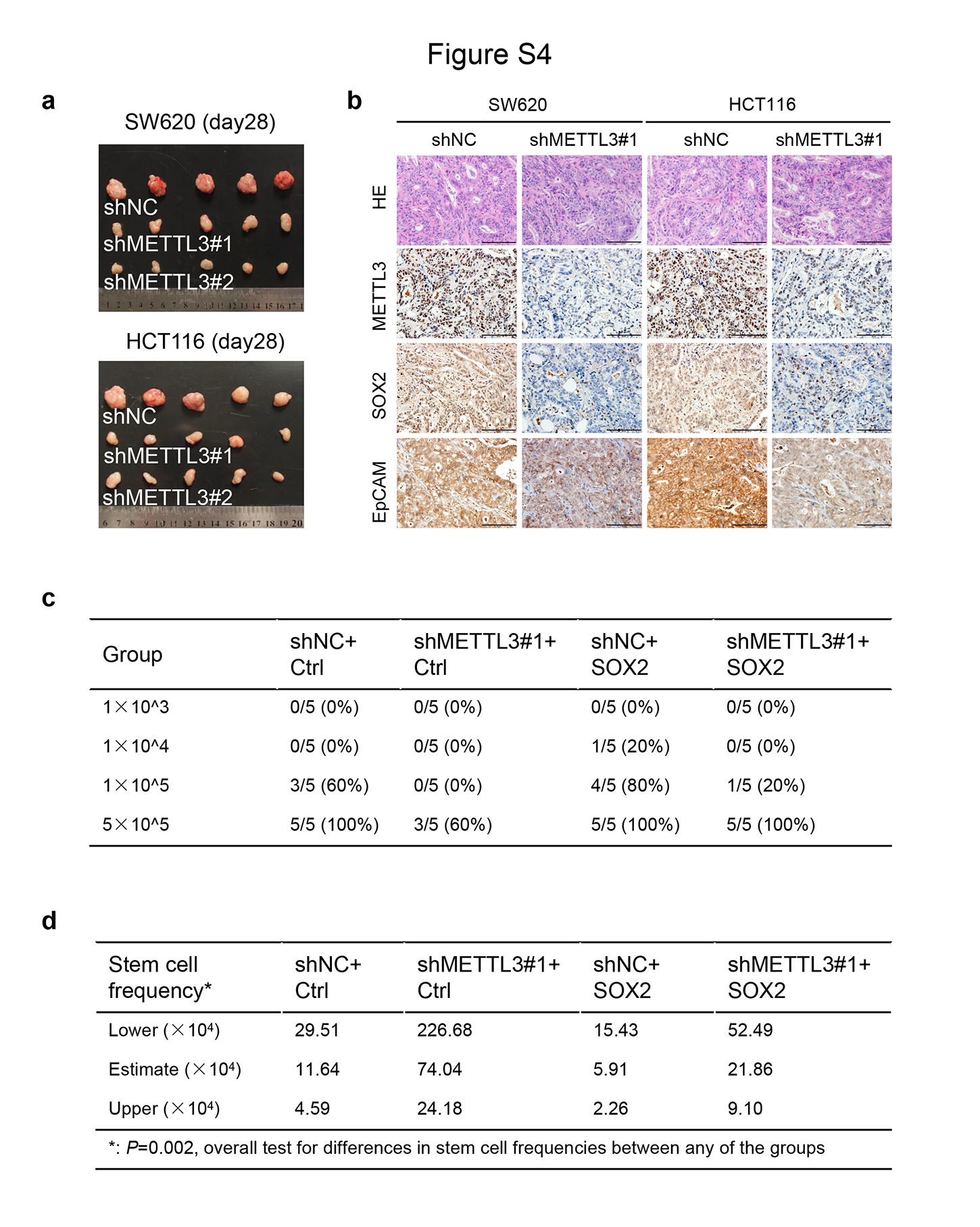

Supplement: Supplementary file 7 — Figure S4, related to Fig. 4: METTL3 drives CRC tumorigenesis and metastasis in vivo. a, Subcutaneous tumor models in nude mice showing the tumor size at day 28 after the implantation of METTL3-knockdown and control SW620 and HCT116 cells (n = 5 mice per group). b, Representative and quantification of H&E and immunostaining (scale bar: 100 μm) of METTL3, SOX2, and EpCAM in subcutaneous tumor models of METTL3 knockdown and control SW620 and HCT116 cells. c, Tumor incidence showing the tumorigenesis of the indicated serial of cell numbers of METTL3 knockdown and control SW620 cells with or without SOX2 overexpression. d, Stem cell frequencies of METTL3 knockdown and control SW620 cells with or without SOX2 overexpression. Estimate: 1/ (the estimated stem cell frequency); Lower, Upper: 95% confidence intervals. All data and error bars are presented as the mean ± SDs. *P < 0.05, **P < 0.01 (Student’s t-test). (TIF 9763 kb) [file 12943_2019_1038_MOESM7_ESM.tif]

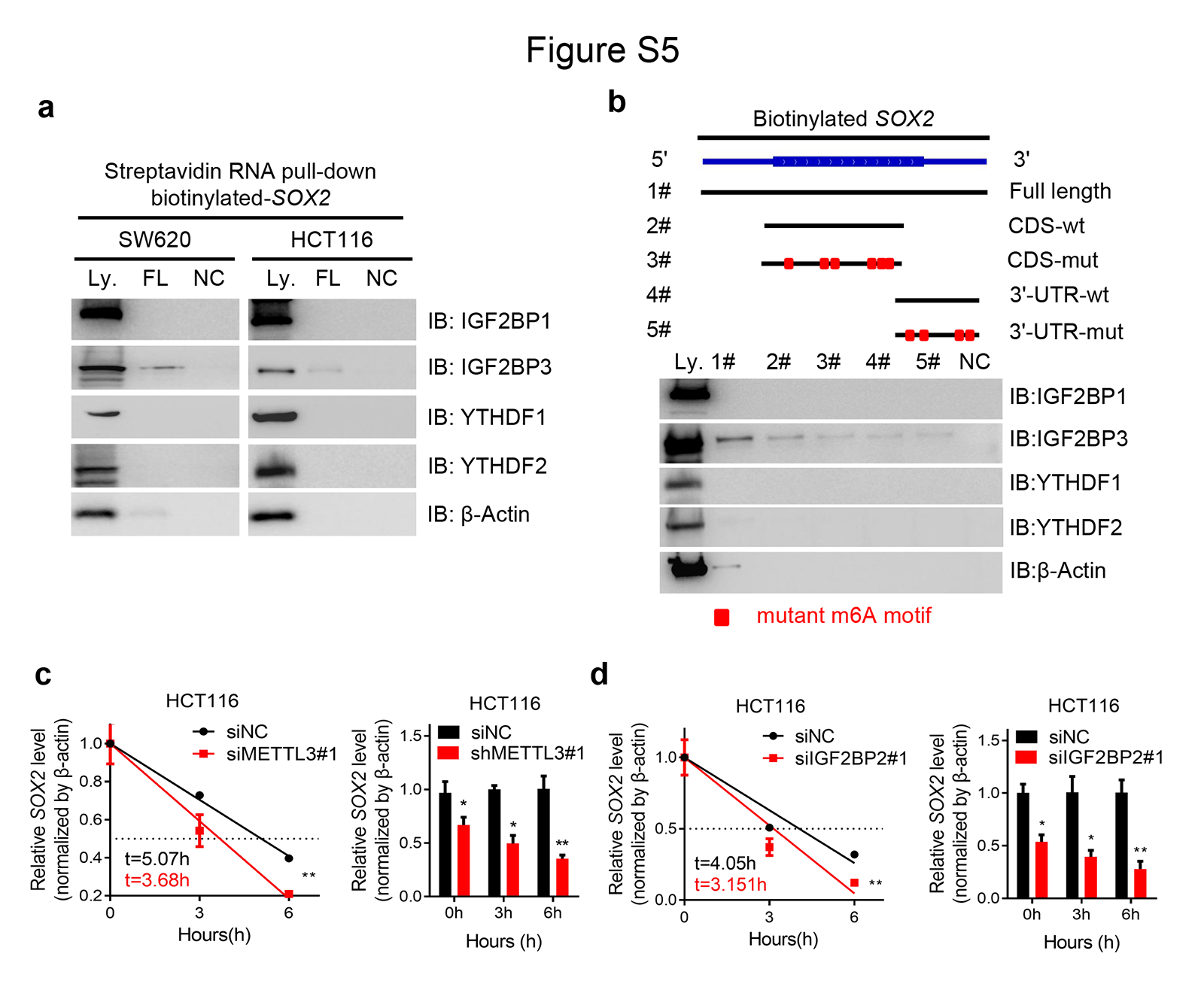

Supplement: Supplementary file 8 — Figure S5 related to Fig. 5: IGF2BP2 enhances SOX2 mRNA stability via an m6A-dependent manner. a, Immunoblotting of IGF2BP1, IGF2BP3, YTHDF1, YTHDF2 after RNA pull down assay with cell lysate (Ly.), full-length biotinylated-SOX2 (FL), and beads only (NC) in SW620 and HCT116 cells. b, Immunoblotting of IGF2BP1, IGF2BP3, YTHDF1, and YTHDF2 with cell lysate (Ly.), full-length biotinylated-SOX2 (#1), the SOX2 CDS region with or without m6A motif mutation (#2, #3), the SOX2 3′-UTR region with or without m6A motif mutation (#4, #5), and beads only (NC) in SW620 cells. c-d, The decay rate of mRNA and qPCR analysis of SOX2 at indicated time after actinomycin D (5 μg/ml) treatment in HCT116 cells after METTL3 inhibition (left), and in HCT116 cells after IGF2BP2 inhibition (right). The date in c, and d are presented as the mean ± SDs (n = 3). *P < 0.05, **P < 0.01 (Student’s t-test). β-Actin was used as the negative control. The relative expression level was normalized by β-Actin. (TIF 7319 kb) [file 12943_2019_1038_MOESM8_ESM.tif]

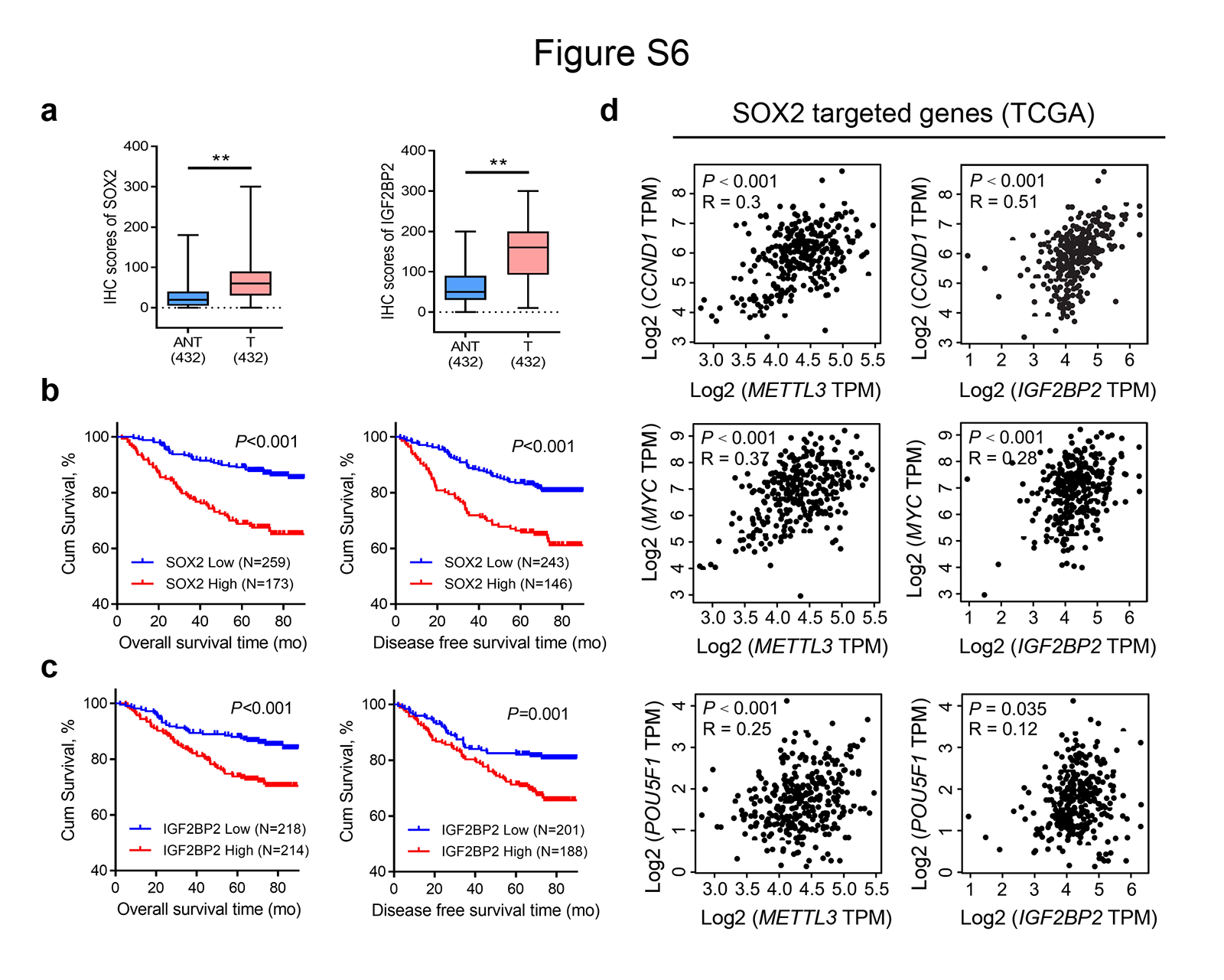

Supplement: Supplementary file 9 — Figure S6 related to Fig. 6: Clinical correlation between METTL3, SOX2 and IGF2BP2 in CRC. a, SOX2 and IGF2BP2 IHC staining scores in primary CRC tumor tissues (T) and adjacent normal tissue (ANT) (n = 432). b-c, Kaplan-Meier analysis of OS and DFS curves based on the expression of SOX2 and IGF2BP2 expression (Kaplan-Meier analysis with the log-rank test). d, Correlation between METTL3 level (left) or IGF2BP2 level (right) with SOX2 target genes, including CCND1, MYC, and POU5F1, in TCGA database for COAD. *P < 0.05, **P < 0.01 (Student’s t-test). (TIF 6918 kb) [file 12943_2019_1038_MOESM9_ESM.tif]
