# Supplementary material for: METTL3 facilitates tumor progression via an m6A-IGF2BP2-dependent mechanism in colorectal carcinoma
Source: Mol Cancer. 2019 Jun 24;18:112. doi: 10.1186/s12943-019-1038-7 (PMC6589893; doi:10.1186/s12943-019-1038-7)
Supplement: Supplementary file 10 — Table S3 Univariate and multivariate analyses of prognostic factors for overall survival among 432 colorectal cancer patients. (DOCX 13 kb) [file 12943_2019_1038_MOESM10_ESM.docx]

**Table S3. The specific sequence of wide-type or m^6^A motif depletion *SOX2* CDS and 3’-UTR.**

| Vectors | Sequence* |
| --- | --- |
| *SOX2 CDS wide-type* | ......GAGTGG**AAACT**TTTGTCG......AGCGCAT**GGACA**GTTACGCGCACATGAACGGCTGGAGCAACGGCAGCTACAGCATGATGCA**GGACC**AGCTGG......CCAGGCCGG**GGACC**TCCG**GGACA**TGATCAGCATGTATCTCCCCGGCGCCGAGGTGCCGGAACCCGCCGCCCCCAGC**AGACT**TCACATGTCCCAGCACTACCAGAGCGGCCCGGTGCCCGGCACGGCCATTAACGGCACACTGCCCCTCTCACACATGTGA |
| *SOX2* CDS mutation | ......GAGTGG**~~AAACT~~**TTTGTCG......AGCGCAT**~~GGACA~~**GTTACGCGCACATGAACGGCTGGAGCAACGGCAGCTACAGCATGATGCA**~~GGACC~~**AGCTGG......CCAGGCCGG**~~GGACC~~**TCCG**~~GGACA~~**TGATCAGCATGTATCTCCCCGGCGCCGAGGTGCCGGAACCCGCCGCCCCCAGC~~AGACT~~TCACATGTCCCAGCACTACCAGAGCGGCCCGGTGCCCGGCACGGCCATTAACGGCACACTGCCCCTCTCACACATGTGA |
| *SOX2* 3’-UTR wide-type | GGGCC**GGACA**GC**GAACT**GGAGG......GACAAGA**AAACT**TTTATGAGAGAGATCCT**GGACT**TCTTTTTGGG**GGACT**ATTTTTGTACAGAGAAAACCTGGGGAGGGTGGGGAGGGCGGGGGAAT**GGACC**TTGTATA......CTGCAGCTGAAATTTA**GGACA**GTTGCAAACGTGAAAAGAAGAAAATTATTCAAATTT**GGACA**TTTTA...... |
| *SOX2* 3’-UTR mutation | GGGCC**~~GGACA~~**GC**~~GAACT~~**GGAGG......GACAAGA**~~AAACT~~**TTTATGAGAGAGATCCT**~~GGACT~~**TCTTTTTGGG**~~GGACT~~**ATTTTTGTACAGAGAAAACCTGGGGAGGGTGGGGAGGGCGGGGGAAT**~~GGACC~~**TTGTATA......GAGAGGCTTCTTGCTGAATTTTGATTCTGCAGCTGAAATTTA**~~GGACA~~**GTTGCAAACGTGAAAAGAAGAAAATTATTCAAATTT**~~GGACA~~**TTTTA....... |
| *The indicated sequence was cloned to the vectors, and the m^6^A motif sites were highlighted. | |
